# Enhanced power grid performance through Gorilla Troops Algorithm-guided thyristor controlled series capacitors allocation

**DOI:** 10.1016/j.heliyon.2024.e34326

**Published:** 2024-07-10

**Authors:** Mohammed H. Alqahtani, Sulaiman Z. Almutairi, Ali S. Aljumah, Ahmed R. Ginidi, Abdullah M. Shaheen

**Affiliations:** a Department of Electrical Engineering, College of Engineering, Prince Sattam bin Abdulaziz Universi-ty, Al Kharj, 16278, Saudi Arabia; b Department of Electrical Engineering, Faculty of Engineering, Suez University, P.O. Box: 43221, Suez, Egypt

**Keywords:** Gorilla troops algorithm, Fitness-based crossover strategy, CEC 2017 benchmark models, TCSC technology, Power grids

## Abstract

This article introduces an innovative application of the Enhanced Gorilla Troops Algorithm (EGTA) in addressing engineering challenges related to the allocation of Thyristor Controlled Series Capacitors (TCSC) in power grids. Drawing inspiration from gorilla group behaviors, EGTA incorporates various methods, such as relocation to new areas, movement towards other gorillas, migration to specific locations, following the silverback, and engaging in competitive interactions for adult females. Enhancements to EGTA involve support for the exploitation and the exploration, respectively, through two additional strategies of periodic Tangent Flight Operator (TFO), and Fitness-based Crossover Strategy (FCS). The paper initially evaluates the effectiveness of EGTA by comparing it to the original GTA using numerical CEC 2017 single-objective benchmarks. Additionally, various recent optimizers are scrutinized. Subsequently, the suitability of the proposed EGTA for the allocation of TCSC apparatuses in transmission power systems is assessed through simulations on two IEEE power grids of 30 and 57 buses, employing various TCSC apparatus quantities. A comprehensive comparison is conducted between EGTA, GTA, and several other prevalent techniques in the literature for all applications. According to the average attained losses, the presented EGTA displays notable reductions in power losses for both the first and second systems when compared to the original GTA. Specifically, for the first system, the proposed EGTA achieves reductions of 1.659 %, 2.545 %, and 4.6 % when optimizing one, two, and three TCSC apparatuses, respectively. Similarly, in the second system, the suggested EGTA achieves reductions of 6.096 %, 7.107 %, and 4.62 %, respectively, when compared to the original GTA's findings considering one, two, and three TCSC apparatuses. The findings underscore the superior effectiveness and efficiency of the proposed EGTA over both the original GTA and several other contemporary systems.

## Highlights

• An enhanced version of GTA, EGTA including FCS and TFO is described.
• An EGTA is proposed for TCSC allocation in power grids.
• Simulations on IEEE power systems have led to significant advancements in energy efficiency.
• It effectively handles TCSC allocation with varying numbers of TCSC apparatuses.
• Demonstrated superior effectiveness of EGTA over GTA and other recent algorithms.

**Table:** Nomenclature (List of variables)

X_TCSC_	TCSC Compensator injected a variable capacitive reactance
α	Controlled thyristors' angle
X_Line_	Transmission-line reactance
OBV	Objective function
θ_uz_ and V_uz_	Disparity in phase angle and voltage among buses u and z
Z_b_	Number of buses
G_uz_	Conductance of the transmission line linking buses u and z
N_lines_	Number of transmission lines
N_TCSC_	Number of TCSC apparatus that will be established
$X_{Line_{TCSC,k}}$	Reactance of the corresponding lines that chosen for TCSC apparatus installation
Line_TCSC_	Potential lines for establishing TCSC apparatus
Ng, Nq, and Nt	Number of generation units, var sources, and transformers, respectively
Pg	Electrical output of generators
Tp	Tap values with reference to tap transformers
QI	Injected electrical power of the var sources
Vg	Generators' voltages
SF	Transmission flow constraints
Qg	Reactive power produced by the generators
Ɠ	Gorilla location solution in the present iteration
Ɠnew	Potential gorilla location vector in the next iterations
m_i_	Arbitrary numbers within a spectrum ranging from 0 to 1 (i = 1,2 … 7)
Ɠ_r_	An individual gorilla that can be determined in any order
Lw and Ur	Upper and lower boundaries of the decision variables
T and MT	Optimization process's maximum and existing iteration counts
ls	An arbitrary number in the interval [-1, 1]
Z	A randomised number in the interval [-C, C]
W	Specified factor
Ɠ_Bt_	The silverback gorilla which is the overall best position
GN	Number of gorillas within the population
L	An adaptive parametr
β	A predefined parameter setting
Ɠ_R1_ and Ɠ_R2_	Two randomly chosen gorillas from the population
By	A binary variable of two possible values: zero and one
σ	A randomly and uniformly distributed value within the band [0, 1]

## 1 Introduction

A static non-linear programming issue that takes into consideration the electrical elements of massive transmission power grids is called the optimal power flow (OPF). While optimizing vital objectives, the primary purpose of the challenge is to identify the steady-state functioning points of all electric elements accessible to the power systems [1,2]. The OPF problem takes into account several individual goals, including entire power losses, the fuel costs for power generation electricity, voltage deviations, polluted emissions, and voltage stability index [3]. Furthermore, the issue of OPF requires that a set of operational and physical constraints be accurately met. These constraints include those enforced by devices and network limitations, such as switchable capacitor banks, transmission line capacity limits, bus voltages, transformer taps, active and reactive generators’ power, and transformer taps [4]. Essentially, in order to obtain other dependent variables such as the voltage magnitude at other buses, and the reactive power of the generators, the control variables such as voltage magnitude at generation buses, the active power of the generators, transformer tap settings, and injected reactive power at capacitor buses of the OPF problem must first be determined [5]. However, the development of Flexible Alternating Current Transmission Systems (FACTs) provides several controllable compensators with additional capabilities to enhance the power grids operation [6].

The utilization of FACTs devices in power systems enhances the static security of a power system. Numerous machine learning approaches have been researched to produce assessments that are both quick and accurate enough where a number of sensitivity and optimization techniques have been reported for appropriate placement and sizing in order to guarantee the efficacy of FACTs devices [7]. Although there hasn't been much use of FACTs in reactive power settings up until recently, there has been a resurgence of interest in this field of study due to recent advances in science [8]. Keeping these things in mind, this study provides up-to-date information on how to enhance the power grids operation by optimally allocating FACTs devices into account. TCSC technology is widely employed in real-world electrical networks as a strong and affordable series FACTS device with outstanding performance that permits accurate reliable power flow control of power lines [9]. One of the most economical ways to free up the transmission network's capacity to carry more real power is to implement TCSC devices, which offers series compensating features [10,11]. Three series FACTS apparatuses of static synchronous series compensator (SSSC), TCSC, and thyristor controlled phase shifter (TCPS) are being taken into consideration and simulated in enhancing the control in multi-area connected electrical networks in order to minimize area frequency fluctuations and tie-line power [12]. The damper controllers were created by combining the Integral of Time multi-plied Squared Error (ITSE) as minimization goal and the Improved Particle Swarm Technique (IPST) as a solution tool. The presented TCSC-AGC outperformed TCPS and SSSC in terms of tie-line transmission powers and vibration dampening at area frequencies. Furthermore, sensitivity testing has been conducted to illustrate the robustness of the TCSC-AGC. This result illustrated the significance and advantageous features of the TCSC over SSSC in transmission networks, indicating its practical application. Academic researchers have recently established a range of traditional and metaheuristic approaches of dealing with OPF [13]. Among the traditional approaches are, gradient approaches, sequential unconstrained methodology [14], interior point method [15], linear and nonlinear programming [16,17], Newton-based method [18], and fuzzy linear methods [19]. It should be mentioned, though, that these techniques do not produce globally optimal solutions and are not beneficial to large electrical networks. Consequently, in an effort to overcome the shortcomings of earlier approaches, scientists have worked to develop metaheuristic methodologies [20]. Even so, these traditional approaches could get stuck in a local optimum since they are dependent on the initial setup and cannot produce the true optimal outcome. Furthermore, each method cannot easily handle integer and discrete variables, where it requires the modelling of particular OPF variations. Consequently, developing metaheuristic techniques is essential to overcoming the above-described limitations [21].

The power system operation in an economical and voltage-secure manner requires the resolution of complex optimization issues requiring advanced computational techniques. Evolutionary computation is a particular approach that has demonstrated its potential to resolve intricate issues. A myriad of population-based methods such as Equilibrium Optimizer (EO) [22], mayfly optimization algorithm [23], jellyfish optimization algorithm [24], artificial rabbits optimization [25], Teaching-Learning Based Optimization (TLBO) [26,27], moth flame combined with quantum computing technique [28], gradient-based optimization algorithm [29], hybridized TLBO method and artificial bee colony [30], combined Arithmetic Optimization Approach (AOA) and aquila optimization (AQUO) [31], and hybrid optimization algorithm of backtracking search with grey wolf techniques [32] are employed to address the OPF. In addition, in Ref. [33], the Whale optimizer, discussed in Ref. [34], was harnessed to pinpoint optimal placements for FACTS in a power network, while concurrently minimizing operational expenses. This optimization version strategically placed TCSC apparatuses initially on weak lines, identified by the L-index indicating instability. The incorporation of TCSC apparatuses into the transmission system, enhancing corresponding available transfer capability, was also investigated in Ref. [35]. In this study, TCSC apparatus locations were predetermined based on the AC power distribution factor (ACPTDF). Subsequently, TLBO was employed to ascertain the pertinent compensation reactance values. Meanwhile, the Gorilla Troops Algorithm (GTA) was previously applied to OPF with TCSC modules in Ref. [36], albeit without due consideration for the allocations of the TCSC. In a distinct approach, a modified crow search technique, detailed in Ref. [37], was employed for OPF with adjustments that amalgamate an innovative bat strategy. Furthermore, the integration of a TCSC apparatus into the electrical system to enhance angle stability, using a PID control system with a filtered component, was explored in Ref. [38]. This study introduced a novel combination of sine cosine optimizer and evolutionary programming to derive optimal stabilizer settings, considering diverse operating conditions and the established power system model featuring a single-machine infinite-bus. Additionally, a specialized EO version has been devised for TCSC apparatus allocation in power systems, aiming to minimize voltage deviations, overload, and losses [39]. A modified NSGA-III with constraint management, outlined in Ref. [40], was introduced to minimize energy loss, featuring an environmental selecting procedure and reduced selecting tries. Despite the commendable aspects of these research papers in addressing OPF, it's noteworthy that none of the mentioned methodologies have taken into account the critical aspects of TCSC size and allocation.

In recent years, the development and refinement of optimization algorithms have become crucial for solving complex engineering and scientific problems. These advancements are evident across various domains, including control systems, power systems, and global optimization challenges. Several innovative methods have demonstrated significant improvements in achieving high accuracy and faster convergence rates including Information-Exchanged Gaussian AOA (IEG-AOA) [41], hybrid Symbiotic Organisms Searching and Simulated Annealing (hSOS-SA) [42] and modified Salp Swarm Optimizer (mSSO) [43], improved SOS (ISOS) [44], Honey Badger Algorithm (HBA) [45], enhanced Marine Predator Algorithm (MPA) [46], Wild Horse Optimization (WHO) [47,48], GTA [49] MPA [50], Firefly Optimization (FO) [51], Fractional-order Kepler Optimizer (FoKO) [52,53], and Cuckoo Search Optimizer (CSO) [54,55], Fractional Hierarchical Gradient Descent (FHGD) [56] and Enhanced Fractional Derivative least mean square (EFDLMS) [57,58]. Table 1 describes some examples of the recent applied algorithms, its key modifications and validation frameworks. These applications highlight the potential for improved convergence and steady-state performance in system identification and optimization. These diverse yet interconnected advancements underscore the pivotal role of optimization algorithms in advancing technological and scientific frontiers.

**Table 1:** Recent applied algorithms, its key modifications and validation frameworks.

Reference	Algorithm	Key Modifications	Applications/Validation
[41]	IEG-AOA	Information exchange among search agents is added while Gaussian distribution to explore promising solutions and Quasi-opposition learning are activated.	23 benchmark functions, 10 CEC2020 test functions, 1 real-life problem
[42]	hSOS-SA	Hybridization of SOS with simulated annealing is presented	Proportional Integral Derivative (PID) controller design for Automatic Voltage Regulator
[43]	mSSO	Sinusoidal map for parameter variation, mutualistic relationship among leader salps and random technique for follower salps are adopted.	Benchmark problems
[44]	ISOS	Quasi-oppositional based learning, enhanced parasitism strategies and Chaotic local search are integrated.	26 benchmark functions, 3 engineering design problems
[45]	HBA	HBA is designed with mixed-variable mathematical modeling	Generation Expansion considerin real sites in Egypt at Zafaranh and Shark El-ouinate.
[46]	Enhanced MPA	It enhanced the predator's strategies by incorporating the potential fluctuations in climate and environmental conditions	Modelling of Kokam lithium-Ion batteries
[47]	WHO	WHO is adopted for designing intelligent fuzzy tilt integral derivative controller	Renewable-dominated micro-grid
[48]		WHO is designed for tuning Fractional-order proportional derivative controller	IEEE 39 power system
[49]	GTA	Artificial GTA is adopted for designing Fractional order PID controller	Interconnected power system
[50]	MPA	MPA is designed for tuning One plus proportional derivative with filter controller	Microgrid system, IEEE 39 test bus validation
[51]	FO	Firefly algorithm for parameter estimation is performed under various noise levels	Power system harmonics analysis
[52]	FoKO	A fractional order component and a local escaping strategy are involved in KO algorithm	CEC 2017 benchmarks with application for IEEE 33 and 69-nodes distribution systems
[53]		A FO-KO algorithm is utilized for tuning a (PI–(1+DD) controller for frequency stability control	CEC 2020 suite and two-area thermal and wind power system
[54,55]	CSO	Swarm intelligence-based optimization Robustness against different SNRs [59]	Power system harmonics, parameter estimation
[56]	FHGD	Fractional hierarchical gradient descent and fractional order adaptation are utilized.	Nonlinear system identification, convergence analysis
[57]	EFDLMS	Enhanced fractional derivative based LMS is introduced while it was effectively applied in Ref. [58] as well.	Parameter estimation, noise analysis

Recently introduced by Abdollahzadeh et al. [60], the GTA is an innovative optimization approach inspired by the social behaviors of gorilla groups. Emphasizing the hierarchical structure led by a silverback gorilla, GTA replicates key aspects of gorilla life, incorporating exploration strategies like migration to unknown and known locations, and interactions with other gorillas. During the exploitation phase, the algorithm mimics behaviors such as following the silverback and engaging in competitive interactions for adult females [61]. GTA is developed based on these observations [62]. The objective of this method is to handle optimization issues in an economical manner while demonstrating great performance and adaptability in different fields. In engineering applications, GTA is user-friendly because it has few configurable parameters. Numerous engineering problems have been successfully tackled by it, such as the OPF issue in electricity energy systems [63], frequency control in virtual power plants [64], bidding system for social welfare maximisation in competitive power systems integrating wind farms [19], coordination of directional overload relays in power systems [65], determination of fuel-cell parameter estimation [66], and frequency control of microgrids [67]. Not only that, but in Ref. [68], a quantum version of a GTA was presented and combined with a quantum-inspired evolutionary algorithm that drew inspiration from the idea of quantum computing [69]. This quantum framework was used to adjust the Tilted-Integral-Derivative controller's settings in a variety of power system stabilizers. It included superposition of quantum states, intervention, and entanglement. To tune PID Controller in micro-robotics apparatuses, a combination method relying on the AOA and GTA was developed in Ref. [70]. In order to improve location update efficiency, a hybridized method centered on the Pelican Optimization Approach (POA) and GTA was created in Ref. [71]. This algorithm combines the predatory qualities of gorillas with the searching abilities of pelicans. The efficacy of this combination strategy was shown in training a Deep Feedforward Neural Network's weights for the purpose of detecting abnormal activity by humans in smart environments.

The allocation of TCSC in power grids is a critical optimization problem with significant implications for improving the efficiency and stability of power systems. Traditional optimization algorithms, including some metaheuristics, often struggle with issues such as premature convergence and getting trapped in local optima. These limitations hinder their effectiveness in solving high-dimensional, complex problems like TCSC allocation. In order to improve upon the GTA, this research suggests an enhanced GTA, EGTA, by merging it with a Fitness-based Crossover Strategy (FCS) and a periodic Tangent Flight Operator (TFO). The EGTA is developed for solving engineering problems and designed for distributing and sizing the apparatuses in power grids for minimizing the whole losses as a crucial objective task. The EGTA is improved by using FCS for promoting its exploration. Contributing to the exploitation step, which entails a recurrent TFO, also solves it. In engineering disciplines, this optimizer is simple to use and has minimal options to change. The following are the primary contributions that this research cites.

• **Enhanced Algorithm Development:** an innovative EGTA including FCS and TFO is presented addressing common metaheuristic challenges such as premature convergence and local optima entrapment.
• **Performance Evaluation:** The proposed EGTA demonstrates significant improvements against GTA and other state-of-the-art algorithms on benchmarking CEC suite and the TCSC allocation problem.
• **Statistical Validation:** Using the Friedman ANOVA test, statistical significance of the results is validated across 28 different functions, confirming the robustness and superiority of EGTA with highly significant p-values.
• **Practical Application:** The proposed EGTA is applied to optimize the allocation of TCSC in power grids, showing its practical utility and effectiveness in solving real-world engineering problems. The proposed EGTA derives significant robustness metrics compared to GTA and other contemporary methods that is previously reported in the literature.

## 2 TCSC allocation in power grids: formulation

### 2.1 TCSC modelling

**Fig. 1:**
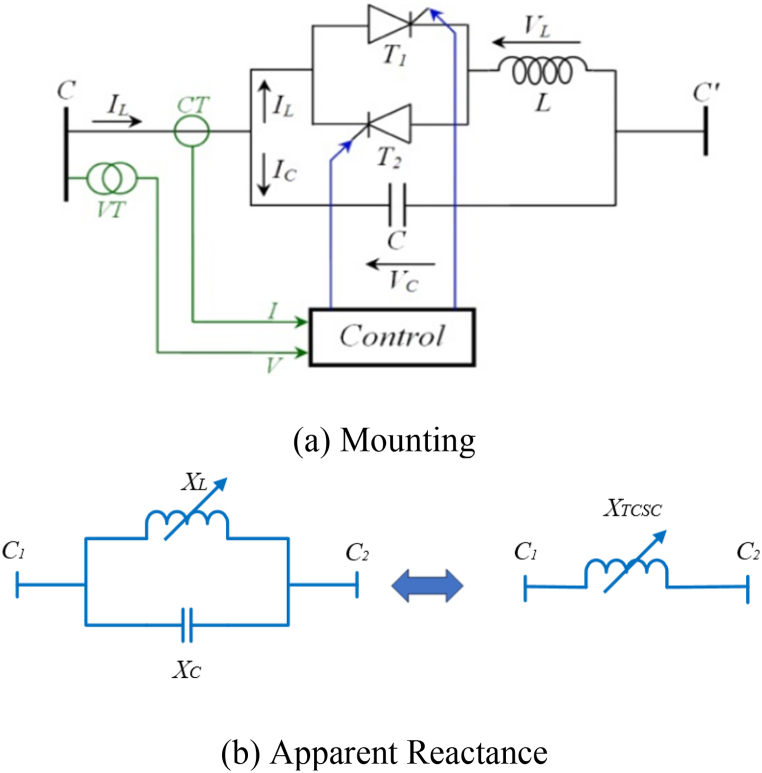
Installed TCSC in series with a line.

The TCSC, a series-type FACTS component with fast reaction times, excellent performance, and low cost, is currently one of the most often utilized elements in the system. TCSC technology can operate in two reactive modes: inductive and capacitive. Hence, it is possible to modify the reactance of the associated line in either an upward or downward direction. It is connected in series with a line as shown in Fig. 1(a). The TCSC is comprised of an inductance (L) controlled by a valve situated between two thyristors (T1 and T2) and a capacitance (C) arranged in parallel [72]. The TCSC, a series-type FACTS component with fast reaction times, The valve's operation is decided by the angle of extinction (α), which can be adjusted to in the range between 90° and 180° [73].

As demonstrated in Fig. 1(b), the compensator TCSC injected a variable capacitive reactance (*X*_*TCSC*_) into the transmission line. The resulting equation determines the controlled thyristors' angle (α). This angle directly impacts the representation of *X*_*TCSC*_ [74,75]. Thus, the TCSC's reactance gets expressed by the transmission-line reactance (*X*_*Line*_). The TCSC apparatus's (X_TCSC_) essential value can be computed by applying Eq. (1) [76,77] in order to stop transmission line overcompensation.

(1) $$X_{TCSC}\left(\alpha \right)=\left(X_{C}\times X_{L}\left(\alpha \right)\right)/\left(X_{C}+X_{L}\left(\alpha \right)\right)$$

(2) $$X_{L}\left(\alpha \right)=-\left(\pi /\left(-\pi +\sin\left(2\alpha \right)+2\alpha \right)\right)X_{L,\max}$$

(3) $$X_{L,\max}=\left(2\pi fL\right);\;X_{C}=-1/j\left(2\pi fC\right)$$

Eq. (1) can be rearranged when replacing the terms *X*_*L*_*(α)* and *X*_*C*_ with their values in Eq. (2) and Eq. (3) as follows:

(4) $$X_{TCSC}\left(\alpha \right)=\frac{\pi {X_{C}X}_{L,\max}}{\pi X_{L,\max}-\left(-\pi +\sin\left(2\alpha \right)+2\alpha \right)X_{C}}$$

### 2.2 Losses minimization and constraints of TCSC allocation

The primary goal is to reduce overall losses in order to technically enhance the electrical system performance and the overall voltage profile. This objective (*OBV*) can be computationally expressed as follows [78]:

(5) $$\text{OBV}=\sum_{u=1}^{\text{Zb}}\left(\sum_{\begin{array}{c} z=1\\ u\neq z \end{array}}^{\text{Zb}}G_{uz}({V_{u}}^{2}+{V_{z}}^{2}‐2\times \left(V_{u}V_{z}\;\cos\;\left(\theta_{uz}\right)\right)\right)$$

The TCSC allocation challenge requires the fulfilment of numerous equality constraints and inequalities constraints to the independent as well as dependent variables. As stated in Eqs. (6), (7), the conditions for reactance compensation, independent variables, and TCSC allocations must be fulfilled.

(6) $$-50\%X_{Line_{TCSC,k}}\geq X_{TCSC}{\left(\alpha \right)}_{k}\geq +50\%X_{Line_{TCSC,p}},k=1,2,...N_{TCSC}$$

(7) $$N_{lines}\geq Line_{TCSC,k}\geq 1,k=1,2,...N_{TCSC}$$

Regarding independent variables, reactive power infusion from Var sources, tap adjustments, generator voltage, and the generator output electrical power are all subjected to limitations managed by Eqs. (10), (11), (8), (9).

(8) $$QI_{Vr}^{\min}\leq QI_{Vr}\leq QI_{Vr}^{\max},Vr=1,2,...Nq$$

(9) $$Vg_{m}^{\min}\leq Vg_{m}\leq Vg_{m}^{\max},m=1,2,...Ng$$

(10) $$Pg_{m}^{\min}\leq Pg_{m}\leq Pg_{m}^{\max},m=1,2,...Ng$$

(11) $$Tp_{k}^{\min}\leq Tp_{k}\leq Tp_{k}^{\max},k=1,2,..Nt$$

Furthermore, with regards to dependent variables, limitations pertaining to apparent power flow across the transmission lines, bus voltage, and generator electrical reactive power production are handled by Eqs. (12), (13), (14).

(12) $$V_{m}^{\min}\leq V_{m}\leq V_{m}^{\max},m=1,2,....Nbus$$

(13) $$\left|SF_{L}\right|\leq SFl_{L}^{\max},L=1,2,....N_{lines}$$

(14) $$Qg_{m}^{\min}\leq Qg_{m}\leq Qg_{m}^{\max},m=1,2,...Ng$$

Accordingly, there must be equality constraints maintained in the reactive as well as active loading balance computations at each bus. The completion of the load flow method fully satisfies these constraints.

## 3 Proposed EGTA optimizer for TCSC allocation in power grids

### 3.1 Original GTA

The cooperative behaviours of gorillas constitute an inspiration for the GTA algorithm's optimization procedure [60]. Three alternative approaches are used to investigate the issue space: travelling in the direction of unknown regions, travelling in the direction of various areas, and travelling along a predetermined path. Two strategies are used in the exploitation stage: monitoring the silverback, which acts as the leader, and competing with adult females.

Every gorilla in the exploration phase is viewed as a possible solution, and the most capable gorilla is named the silverback, in charge of the optimization processes. Before the optimization process is initiated, an attribute called “*p*" arbitrarily selected between 0 and 1, determines the migration manner to use. The move to an unidentified place is selected if a randomly selected magnitude, called " *m*_*2*_,” has a lower than the attribute “*p*". The practice of approaching nearby gorillas is favoured, though, if " *m*_*2*_″ is more than “*p*" and surpasses 0.5. Finally, the migration in the direction of a specific site is chosen if “*m*_*4*_″ is larger than “*p*" yet below 0.5. Equation (15) using can be used to explain the different exploration strategies.

(15) $$Ɠnew=\left\{ \begin{array}{c} Lw+\left(Ur-Lw\right)\times m_{1},f\;m_{2}<p\;\\ \begin{array}{c} \left(m_{3}-C\right)\times {Ɠ}_{r}+L\times Z\times Ɠ,If\;m_{4}\geq 0.5,\\ Ɠ+L^{2}\times \left(\left({Ɠ}_{r}-Ɠ\right)\right)-m_{5}\times \left({Ɠ}_{r}-Ɠ\right),Else \end{array}\}\;Else \end{array}\right.$$

Therefore, the parameters *C*, *Z* and *L* are quantitatively described as:

(16) $$C=\left(\cos\left(2\times m_{6}\right)+1\right)\times \left(1-\frac{T}{MT}\right)$$

(17) Z=[−C,C]

(18) $$L=\left(\cos\left(2\times m_{7}\right)+1\right)\times \left(1-\frac{T}{MT}\right)\times ls$$

Two techniques of responding to the silverback or competing for females were specifically designed for the GTA algorithm's exploitation phase. Choosing which approach to use is determined by contrasting one parameter, represented by Equation (10), with another, represented by *W*. Prior to the optimization procedure, a numerical value for the factor *W* must be supplied. As per reference [60], it has been configured at 0.8.

(19) $$Ɠnew=Ɠ-L\times M_{z}\times \left({Ɠ}_{Bt}-Ɠ\right)$$

(20) $$M_{z}={\left({\left|\sum_{J=1}^{GN}\frac{{Ɠ}_{J}}{N}\right|}^{2^{L}}\right)}^{\left(2^{-L}\right)}$$

If the value of *C* is greater than or equal to *W*, as expressed in Equation (15), the strategy of following the silverback gorilla is activated. At the contrary conjunction, the alternative approach is applied if *C* is less than *W*. When gorillas reach adulthood, they compete fiercely with one another for the attention of females.

Equation (21) is applied in order to replicate the violent character of the competition by simulating the strong influence on the individuals' dimensions.

(21) $$Ɠnew={Ɠ}_{Bt}-\left(\beta \times E\times \left(2m_{8}-1\right)\left({Ɠ}_{Bt}-Ɠ\right)\right)$$

(22) $$E=\left\{ \begin{array}{c} N1\;m_{9}\geq 0.5\\ N2\;m_{9}<0.5 \end{array}\right.$$

The main steps of the original GTA in handling the optimal TCSC allocations in power grids are summarized in the pseudocode 1. As shown, the comparison between C and W in the exploitation portion of the GTA method determines whether to adhere to the silverback or compete for females. When C is larger than or identical to W, the competition approach is engaged; conversely, when C is less than W, the silverback approach is chosen. By introducing dynamics that mimic gorilla population behaviours, these tactics improve the optimization process.

**Table:** 

Pseudocode 1. GTA steps
1. Set parameters: *GN* (population size), *TM* (maximum iterations), *β*, *p*, *W* 2. Insert data of the system under study: Generation data, Lines data and the boundaries of $X_{TCSC}{\left(\alpha \right)}_{k}$ regarding Eq. (6) and $QI_{\text{Vr}},Vg_{m},Pg_{m}and\;Tp_{k}$ regarding Eqs. 8–11 3. Initialize population of gorillas (Ɠ) 4. For each gorilla i in Ɠ: 5. Assign the values of the control variables ($X_{TCSC}{\left(\alpha \right)}_{k}$, $QI_{\text{Vr}},Vg_{m},Pg_{m}and\;Tp_{k}$) 6. Apply Load flow routine to estimate the bus voltages ($V_{m}$), lines flow ($SF_{L}$) and reactive power from generators ($Qg_{m}$) 7. Check their values ($V_{m}$, $SF_{L}$ and $Qg_{m}$) to be inside the permissible limits 8. Evaluate the overall losses (OBV) using Eq. (4) 9. Assign fitness: Fit(Ɠ) as the losses 10. For T = 1 to TM 11. Update C, Z, and L according to Eqs. 16–18 12. For each gorilla i in Ɠ: 13. Generate new position (${Ɠnew}_{i}$) using Eq. (15) 14. Repeat Lines 5:8 and assign the fitness of new position: Fit(${Ɠnew}_{i}$) 15. Replace (${Ɠ}_{i}$) with new position (${Ɠnew}_{i}$) if fitness improves 16. Update ${Ɠ}_{Bt}$ (best solution found so far) 17. For each gorilla i in Ɠ: 18. If C ≥ W: 19. Update position (Ɠnew) using Eq. (19) 20. Else: 21. Update position (Ɠnew) using Eq. (21) (consider ${Ɠ}_{Bt}$, best solution so far) 22. End If 23. Repeat Lines 5:8 and assign the fitness of new position: Fit(${Ɠnew}_{i}$) 24. Update fitness of all gorillas in Ɠ. 25. Update ${Ɠ}_{Bt}$ (best solution found so far) 26. End loop 27. Return ${Ɠ}_{Bt}$ (optimal solution)

### 3.2 Proposed EGTA

This section presents EGTA, an improved version of the GTA optimizer that combines two changes to provide the conventional GTA with more exploratory and exploitation features. A FCS is introduced in the initial modification to support the discovery stage [79,80]. Two randomly chosen gorillas are chosen for every dimension in the suggested FCS, and an alternative solution is produced using the lowest fitness metric. For each dimension, two gorillas are randomly chosen which are *Ɠ*_*R1*_ and *Ɠ*_*R2*_ from the population which its size is typically denoted as “*GN*”. Next, the fitness values of these two randomly chosen gorillas, *Fit*(*Ɠ*_*R1*_) and *Fit*(*Ɠ*_*R2*_), are obtained. These fitness values represent the performance or quality of the solutions provided by the gorillas in the current population. The FCS method then compares the fitness values of *Ɠ*_*R1*_and *Ɠ*_*R2*_ to determine which gorilla has the minimum fitness. The minimum fitness indicates the better-performing solution. Based on the minimum fitness criterion, the FCS method selects an assistant solution vector, denoted as *Ɠd*, as follows:

(23) $${Ɠd}_{k}=\left\{ \begin{array}{c} {Ɠ}_{R1,k}\;If\;Fit\left({Ɠ}_{R1}\right)\leq Fit\left({Ɠ}_{R2}\right)\\ {Ɠ}_{R2,k}\;If\;Fit\left({Ɠ}_{R1}\right)>Fit\left({Ɠ}_{R2}\right) \end{array}\right.;k=1:\mathrm{Dim}$$

In other words, the position of the gorilla with the better fitness becomes the assistant solution vector. Based on that FCS, a new gorilla position can be generated as follows:

(24) Ɠnew=PR×Ɠd+(1−By)×Ɠ

(25) $$By=\left\{ \begin{array}{c} 1\;If\;m_{10}\leq 0.5\\ 0\;Else \end{array}\right.$$

The present location of the gorilla is replaced with a newly produced seeking vector by utilizing the FCS's suggested method. As a result, the FCS keeps the computation pace constant by maintaining an equivalent amount of function executions. The gorillas' existing locations will be replaced by a set of newly created locations that are produced by repeating this method in every dimension. The FCS technique adds diversity to the discovery process and promotes exploration by choosing two randomly chosen gorillas and assigning the new place to the gorilla with the lowest fitness.

In addition, a periodic tangent function [81] is incorporated for use in the process of exploitation by adding a TFO in the following manner:

(26) $$fl=\tan\left(\sigma \times \frac{\pi }{2}\right)$$

(27) σ=randn(1,Dim)

This approach allows for an efficient investigation of searching area. The TFO is added to Eq. (19) by the suggested EGTA approach. By reducing the distance between the gorilla and the silverback, this modification significantly lowers the end step size and raises the objective rating. The following is a mathematical description of this model:

(28) $$Ɠnew=Ɠ\times \left(\frac{L}{100}\times M_{z}\times \left(1-\frac{{Ɠ}_{Bt}}{Ɠ}\right)\times \tan\;\left(\frac{\pi }{2}\times \left(2\sigma -1\right)\right)+1\right)$$

The key steps of the proposed EGTA in handling the optimal TCSC allocations in power grids are summarized in the pseudocode 2. This provided pseudocode effectively encapsulates the core concepts, with clear steps for initialization, fitness evaluation, and iterative optimization. The incorporation of TFO to improve convergence and precision is a notable enhancement. It can be summarized in four main steps.

Step 1 Initialization: It includes parameter setup, system data input and creation of the initial population of gorillas with random positions within specified bounds. Also, the fitness score for each solution is evaluated.

Step 2 Main Loop for Iterations: It involves parameters update (C, Z, and L) and the application of exploration and exploitation techniques. Also, the fitness score for each new generated solution is evaluated. In this step, the exploitation is activated if the new position does not provide better fitness by selecting two random gorillas and generating a new position based on the one with lower fitness using FCS.

Step 3 Follow or Compete: If the parameter C is greater than or equal to W, the gorilla's position is updated by following the best solution found so far. On the other side, if C is less than W, the TFO is applied to refine the position updates, promoting more precise exploration and improved convergence towards the optimal solution.

Step 4 Final Updates and Return: It includes the fitness and best solution update and returning the optimal solution.

**Table:** 

Pseudocode 2. Proposed EGTA steps
1. Set parameters: *GN* (population size), *TM* (maximum iterations), *β*, *p*, *W* 2. Insert data of the system under study: Generation data, Lines data and the boundaries of $X_{TCSC}{\left(\alpha \right)}_{k}$ regarding Eq. (6) and $QI_{\text{Vr}},Vg_{m},Pg_{m}and\;Tp_{k}$ regarding Eqs. 8–11 3. Initialize population of gorillas (Ɠ) 4. For each gorilla i in Ɠ: 5. Assign the values of the control variables ($X_{TCSC}{\left(\alpha \right)}_{k}$, $QI_{\text{Vr}},Vg_{m},Pg_{m}and\;Tp_{k}$) 6. Apply Load flow routine to estimate the bus voltages ($V_{m}$), lines flow ($SF_{L}$) and reactive power from generators ($Qg_{m}$) 7. Check their values ($V_{m}$, $SF_{L}$ and $Qg_{m}$) to be inside the permissible limits 8. Evaluate the overall losses (OBV) using Eq. (4) 9. Assign fitness: Fit(Ɠ) as the losses 10. For T = 1 to TM 11. Update C, Z, and L according to Eqs. 16–18 12. For each gorilla i in Ɠ: 13. If a random number generated ≤ 0.5 14. Generate new position (Ɠnew) using Eq. (15) 15. Repeat Lines 5:8 and assign the fitness of new position: Fit(${Ɠnew}_{i}$) 16. Replace (${Ɠ}_{i}$) with new position (${Ɠnew}_{i}$) if fitness improves 17. Else 18. Select two random gorillas (${Ɠ}_{R1}$, ${Ɠ}_{R2}$) 19. Ɠd = gorilla with lower fitness (Fit(${Ɠ}_{R1}$), Fit(${Ɠ}_{R2}$)) # Apply FCS using Eq. (23) 20. Generate new position (${Ɠnew}_{i}$) using Eqs. (24), (25) (based on ${Ɠ}_{i}$, Ɠd) 21. Repeat Lines 5:8 and assign the fitness of new position: Fit(${Ɠnew}_{i}$) 22. Replace (${Ɠ}_{i}$) with new position (${Ɠnew}_{i}$) if fitness improves 23. End If 24. Update ${Ɠ}_{Bt}$ (best solution found so far) 25. For each gorilla i: 26. If C ≥ W: # Follow the silverback 27. Update position (Ɠnew) using Eq. (21) (consider ${Ɠ}_{Bt}$, best solution so far) 28. Else: # Compete for females 29. Update σ using Eq. (27) 30. Apply TFO to update the position (Ɠnew) using Eq. (28) (reduce step size, improve convergence) 31. End If 32. Repeat Lines 5:8 and assign the fitness of new position: Fit(${Ɠnew}_{i}$) 33. Update fitness of all gorillas 34. Update ${Ɠ}_{Bt}$ (best solution found so far) 35. End loop 36. Return ${Ɠ}_{Bt}$ (optimal solution)

## 4 Results and discussion

In this section, we delve into the application of the proposed novel EGTA in two distinct dimensions. Firstly, we conduct simulations on benchmark functions, specifically addressing the CEC 2017 benchmarks. These simulations involve a thorough comparison with several recently developed metaheuristic algorithms. Secondly, simulations are extended to tackle TCSC allocation challenges within power networks, focusing on two IEEE original power systems comprising 30 and 57 buses.

### 4.1 Evaluation of application performance using CEC 2017 benchmark models

**Fig. 2:**
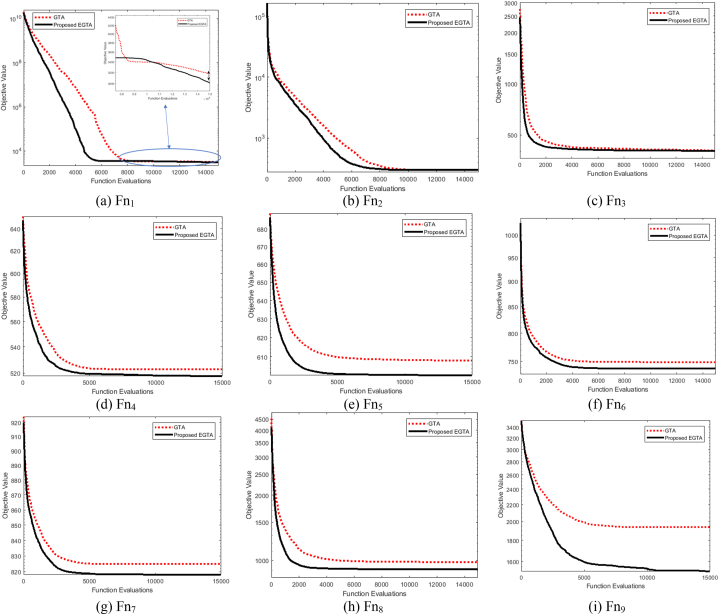

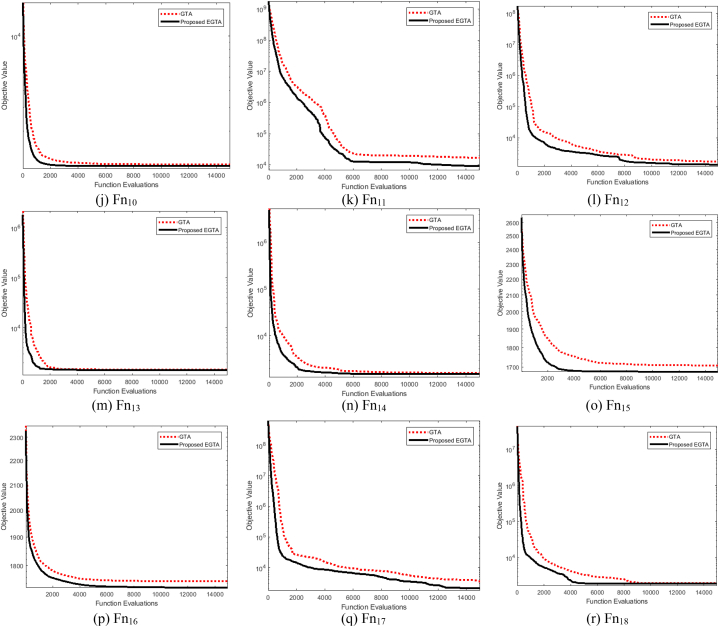

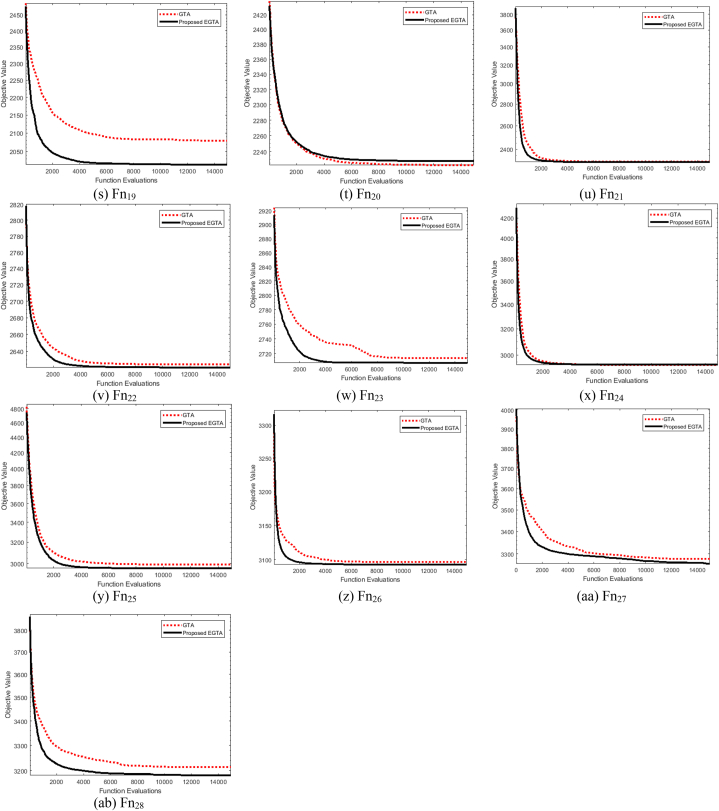
Average convergence properties of EGTA and GTA for CEC 2017 problems.

Ascertaining the effectiveness of optimization strategies can be challenging without a formal proof of their efficacy. Benchmark functions play a crucial role in evaluating the performance of these strategies. In this context, we assess the performance of the proposed EGTA and GTA techniques through the utilization of the CEC 2017 competition as a benchmark [82]. This competition encompasses a variety of routines designed to evaluate different attributes, including unimodal, multimodal, mixed, and composite functions. An overview of the unrestricted benchmarking functions employed in our analysis is described in Ref. [73]. Across all 28 benchmarking functions, we consider a dimension of 30 control variables, with their bounds set at [−100, 100]. The proposed EGTA undergoes evaluation in comparison to the conventional GTA, considering CEC 2017 single-objective optimization. The mean convergence characteristics of EGTA and GTA are visually presented in Fig. 2, providing insights into their performance on CEC 2017 problems. The mean convergence is utilized rather than the best run to provide a more representative and statistically robust comparison of the algorithms' performance. Furthermore, a comprehensive comparison involves several contemporary optimization techniques, namely AQUO [83], red kite optimization (RKO) [84], and subtraction-average-based algorithm Technique (SAT) [85]. The specific settings and a range of successful applications for each contrasted technique are detailed in Table 2. From this table, the number of fitness function evaluations for all algorithms are similar by 15,000 in order to conduct a fair comparison. In this regard, the number of gorillas solutions is fifteen since GTA and EGTA perform two function evaluations per solution which are double the times of the others. Metaheuristic techniques are inherently stochastic, as they rely on generating several random parameters. Because of this stochastic nature, different runs of the same algorithm can start from different initial points, and this variability is a fundamental characteristic of these methods. To ensure a fair and comprehensive comparison, each algorithm was executed independently for 50 runs. The performance was then assessed using statistical measures such as the maximum, mean, minimum, and standard deviation of the results across these runs. Table 2 furnishes statistical metrics, encompassing the best, mean, worst, and standard deviation (Std) outcomes for the compared methods.

**Table 2:** Compared algorithms: Parameters and applications.

Algorithm	Parameters	Number of function evaluations
DMO (2022) [86]	Number of babysitters = 3; Alpha vocalization (peep = 2); Number of dwarfs = 30; maximum iterations number = 500.	30*500 = 15,000
AQUO (2021) [83]	Number of Aquilla's = 30; number of iterations = 500; Delta = Alpha = 0.1.	30*500 = 15,000
GTA (2021) [60]	Number of gorillas = 15; iterations number = 500	15*2*500 = 15,000
Proposed EGTA	Number of gorillas = 15; iterations number = 500	15*2*500 = 15,000
RKO (2022) [84]	Number of searching agents = 30; iterations number = 500	30*500 = 15,000
SAT (2023) [85]	Number of searching agents = 30; iterations number = 500	30*500 = 15,000

Remarkably, as illustrated in Table 3, the EGTA technique exhibits superior efficacy, consistently achieving the lowest statistical indices across the majority of benchmark functions. Moreover, Table 3 provides insights into the outcomes of a Friedman ranking test conducted on the compared algorithms. As evident, the meticulously designed EGTA attains a commendable average rank of 1.848, securing the topmost position. In the subsequent tier, DMO achieves a mean rank of 2.8303, while the original GTA occupies the third spot with a rank of 3.424. Following suit, RKO and SAT claim the fourth and fifth positions, boasting mean ranks of 3.634 and 4.495, respectively. Notably, AQUO lingers at the bottom with a mean rank of 4.767. These results underscore the remarkable performance of EGTA, showcasing improvement reductions of 34.7 %, 46.02 %, 49.14 %, 58.88 %, and 61.2 % compared to DMO, GTA, RKO, SAT, and AQUO, respectively. Such compelling advancements highlight EGTA's efficacy and robustness across diverse optimization scenarios.

**Table 3:** Statistical outcomes of the EGTA compared to GTA, DMO, SAT, RKO and AQUO for CEC 2017 tasks.

		Obtained results						Associated orders					
No.	Index	EGTA	GTA	DMO	SAT	RKO	AQUO	EGTA	GTA	DMO	SAT	RKO	AQUO
*Fn*_*1*_	Best	115.0402	100.0138	103.1033	106.8504	101.3583	631630.4	5	2	3	4	1	6
	Mean	2712.3	2784.152	4947.712	3812.14	6394.494	7117627	1	2	4	3	5	6
	Worst	12586.73	12625.35	63834.88	12866.27	12725.47	47965063	1	2	5	4	3	6
	Std	2634.386	2798.644	12150.27	3324.979	4340.656	8879770	1	3	5	2	4	6
*Fn*_*2*_	Best	300	300	426.9525	433.8029	301.5553	862.8424	1.5	1.5	4	5	3	6
	Mean	300.1742	301.4181	1075.823	1892.281	1791.484	14241.48	1	2	3	5	4	6
	Worst	302.5147	346.8231	2487.439	20047	28092.82	78883.29	1	2	3	4	5	6
	Std	0.511405	6.731783	420.1405	2775.047	4446.282	17008.76	1	2	3	4	5	6
*Fn*_*3*_	Best	400.0073	400.0001	400.5637	403.8382	405.4006	402.9141	2	1	3	5	6	4
	Mean	404.5448	412.5847	405.2926	410.8517	450.0849	418.5362	1	4	2	3	6	5
	Worst	407.2395	478.4354	406.8646	496.1308	905.8536	576.6982	2	3	1	4	6	5
	Std	2.240689	21.25876	1.199943	18.50037	78.84186	29.72566	2	4	1	3	6	5
*Fn*_*4*_	Best	502.9849	503.9798	518.0095	511.9395	502.097	514.84	2	3	6	4	1	5
	Mean	519.9204	529.9719	530.0403	535.7457	516.5253	528.8363	2	4	5	6	1	3
	Worst	544.7729	568.6719	539.6044	577.6057	543.6022	552.2148	3	5	1	6	2	4
	Std	7.548553	12.82694	5.370918	16.49356	10.16285	9.884062	2	5	1	6	4	3
*Fn*_*5*_	Best	600.009	602.4584	600	603.8165	600	607.8623	3	4	1.5	5	1.5	6
	Mean	601.563	614.2151	600	617.543	600.623	616.8517	2	4	1	6	3	5
	Worst	606.9587	646.6092	600.0001	647.1749	617.8881	636.7507	2	4	1	6	3	5
	Std	1.776384	9.243879	1.77E-05	9.427292	2.806871	6.212097	2	5	1	6	3	4
*Fn*_*6*_	Best	717.0478	723.7079	728.6148	721.5113	714.6422	728.4909	2	4	6	3	1	5
	Mean	743.4281	762.1251	743.0721	751.7038	734.864	760.7281	3	6	2	4	1	5
	Worst	776.7059	811.2731	753.9905	791.7054	761.6374	812.3142	3	5	1	4	2	6
	Std	12.36413	20.19273	5.371381	18.37205	12.40402	18.5329	2	6	1	4	3	5
*Fn*_*7*_	Best	802.9849	803.9798	811.2899	805.9697	803.0511	812.173	1	3	5	4	2	6
	Mean	821.3062	825.6383	829.6501	822.3442	813.5723	828.0318	2	4	6	3	1	5
	Worst	836.8134	847.7577	841.7104	841.7882	837.339	846.8473	1	6	3	4	2	5
	Std	7.440052	8.340179	6.106073	7.530569	9.903987	7.133653	3	5	1	4	6	2
*Fn*_*8*_	Best	900	904.9497	900	910.5194	900	935.9179	2	4	2	5	2	6
	Mean	946.883	1038.196	900	1080.856	900.9298	1080.06	3	4	1	6	2	5
	Worst	1150.876	1399.77	900	1895.92	918.829	1358.738	3	4	1	6	2	5
	Std	57.01877	116.6759	2.53E-09	170.4958	2.873668	93.07999	3	4	1	6	2	5
*Fn*_*9*_	Best	1122.103	1503.696	1615.589	1221.682	1382.47	1282.538	1	5	6	2	4	3
	Mean	1669.006	2068.6	2276.483	2024.885	1809.26	1898.988	1	4	6	5	2	3
	Worst	2166.557	2564.761	3533.997	3318.621	2535.195	2405.251	1	4	6	5	3	2
	Std	254.4514	286.3009	277.9387	370.4943	324.4173	239.4575	2	4	3	6	5	1
*Fn*_*10*_	Best	1102.144	1103.108	1103.032	1103.253	1101.18	1126.013	2	4	3	5	1	6
	Mean	1112.653	1138.331	1107.101	1158.702	1152.762	1245.853	2	3	1	5	4	6
	Worst	1134.107	1208.154	1112.527	1273.258	1361.733	1543.506	2	3	1	4	5	6
	Std	7.215987	27.10392	2.507902	43.42037	85.62553	95.9905	2	3	1	4	5	6
*Fn*_*11*_	Best	1499.141	2396.646	7581.318	2220.099	11046.39	59003.65	1	3	4	2	5	6
	Mean	11739.94	22675.91	147990.2	16173.04	556267.5	5839942	1	3	4	2	5	6
	Worst	38550.63	62138.9	848651.8	53781.33	15705336	21294607	1	3	4	2	5	6
	Std	9144.067	16884.75	157984.9	13805.32	2309722	6235390	1	3	4	2	5	6
*Fn*_*12*_	Best	1306.017	1320.665	1471.087	1521.598	1596.162	2983.547	1	2	3	4	5	6
	Mean	2088.816	2125.133	4806.373	11073.28	12489.74	16104.91	1	2	3	4	5	6
	Worst	13442.78	6264.009	14058.03	36272.32	72354.44	57140.58	2	1	3	4	6	5
	Std	2085.528	805.6233	2962.256	9503.086	11704.68	11977.37	2	1	3	4	5	6
*Fn*_*13*_	Best	1402.097	1416.848	1437.532	1451.43	1468.283	1475.86	1	2	3	4	5	6
	Mean	1423.935	1472.577	93986408	3218.386	1594.974	2726.689	1	2	6	5	3	4
	Worst	1450.175	1555.487	3.81E+09	14794.43	2492.51	7300.955	1	2	6	5	3	4
	Std	13.78748	33.27598	5.51E+08	2633.219	161.0686	1181.206	1	2	6	5	3	4
*Fn*_*14*_	Best	1501.297	1520.28	1574.339	1562.425	1666.848	1704.87	1	2	4	3	5	6
	Mean	1519.631	1651.325	1894.31	3284.562	2545.096	7155.795	1	2	3	5	4	6
	Worst	1616.562	2014.949	2742.553	5926.97	4767.804	13013.25	1	2	3	5	4	6
	Std	18.72157	121.4205	289.9846	1216.997	803.5599	2819.584	1	2	3	5	4	6
*Fn*_*15*_	Best	1600.253	1601.517	1601.544	1601.703	1600.854	1625.733	1	3	4	5	2	6
	Mean	1721.43	1719.189	1613.991	1817.625	1632.426	1799.318	3	4	1	6	2	5
	Worst	2004.347	1990.167	1738.793	2135.552	1900.84	2117.308	4	3	1	6	2	5
	Std	118.5516	108.4578	22.69649	142.0484	58.07911	128.3401	4	3	1	6	2	5
*Fn*_*16*_	Best	1701.512	1722.671	1712.647	1723.965	1706.86	1745.723	1	4	3	5	2	6
	Mean	1730.73	1751.659	1740.727	1783.957	1742.974	1781.167	1	3	2	6	4	5
	Worst	1784.029	1805.343	1761.893	1925.583	1781.586	1858.912	3	4	1	6	2	5
	Std	18.8871	17.30333	9.774917	53.54611	11.82739	26.89721	4	3	1	6	2	5
*Fn*_*17*_	Best	1809.14	1937.841	1983.072	1990.645	3489.732	6124.661	1	2	3	4	5	6
	Mean	4170.913	7672.947	7055.869	10866.06	33008.39	45811.23	1	3	2	4	5	6
	Worst	17244.14	34696.87	19019.33	55426.05	209371	72733.15	1	3	2	4	6	5
	Std	3387.31	7723.696	4063.737	11000.98	34519.64	15609.74	1	3	2	4	6	5
*Fn*_*18*_	Best	1900.58	1907.628	1911.531	2025.102	1947.243	2007.858	1	2	3	6	4	5
	Mean	1912.763	1996.652	2172.458	11852.15	3129.286	25488.55	1	2	3	5	4	6
	Worst	2007.547	2378.405	3884.533	208941.3	9117.7	120047.6	1	2	3	6	4	5
	Std	22.03206	88.11529	328.5189	28897.4	1670.582	21289.85	1	2	3	6	4	5
*Fn*_*19*_	Best	2000.624	2020.994	2000.323	2031.366	2001.065	2049.27	1	4	2	5	3	6
	Mean	2025.766	2107.056	2010.275	2146.249	2040.972	2134.692	2	4	1	6	3	5
	Worst	2131.493	2238.633	2035.596	2324.983	2161.175	2262.899	2	4	1	6	3	5
	Std	24.87773	61.06534	10.40192	74.54465	48.08278	56.72375	2	4	1	6	3	5
*Fn*_*20*_	Best	2200	2200.003	2207.963	2200	2202.68	2206.064	1.5	3	6	1.5	4	5
	Mean	2232.667	2234.167	2421.83	2311.109	2269.041	2303.841	1	2	6	5	3	4
	Worst	2328.089	2350.29	3423.376	2379.046	2341.668	2348.035	1	4	6	5	2	3
	Std	52.53154	54.78871	276.8203	53.57661	59.03082	49.8612	2	4	6	3	5	1
*Fn*_*21*_	Best	2215.56	2300.822	2290.772	2244.561	2225.953	2245.189	1	6	5	3	2	4
	Mean	2304.241	2309.889	2302.682	2309.082	2395.433	2310.746	2	4	1	3	6	5
	Worst	2323.311	2352.651	2306.795	2345.843	3882.778	2331.133	2	5	1	4	6	3
	Std	13.52277	10.26236	2.544353	12.51222	333.9873	14.20948	4	2	1	3	6	5
*Fn*_*22*_	Best	2606.818	2605.333	2611.39	2614.191	2605.674	2614.735	3	1	4	5	2	6
	Mean	2623.618	2633.175	2626.133	2655.502	2628.164	2639.714	1	3	2	6	4	5
	Worst	2641.827	2745.878	2643.387	2714.223	2712.256	2680.313	1	3	2	6	5	4
	Std	7.973903	23.27746	7.061391	25.23442	25.15645	13.18305	2	4	1	6	5	3
*Fn*_*23*_	Best	2500	2500.001	2529.358	2500	2737.125	2743.333	1.5	3	4	1.5	5	6
	Mean	2740.001	2756.561	2779.46	2776.754	2760.733	2765.077	1	2	6	5	3	4
	Worst	2781.422	2850.976	4667.793	2881.822	2862.941	2791.924	1	3	6	5	4	2
	Std	62.08293	41.08048	403.3049	48.65351	25.74767	11.60206	5	3	6	4	2	1
*Fn*_*24*_	Best	2897.743	2897.94	2898.008	2897.941	2897.94	2898.884	1	2	5	4	3	6
	Mean	2925.564	2941.376	2923.861	2927.008	2928.859	2938.885	2	6	1	3	4	5
	Worst	2951.323	3024.373	2945.222	2978.515	2953.657	3030.515	2	6	1	4	3	5
	Std	23.38237	24.13621	20.46398	25.58298	24.11888	27.90755	2	4	1	5	3	6
*Fn*_*25*_	Best	2600	2800	2606.09	2600	2900	2825.635	1.5	4	3	1.5	6	5
	Mean	2976.738	3057.343	2865.906	3132.256	3165.254	3020.676	2	3	1	5	6	4
	Worst	3335.847	3989.706	2900.003	4234.516	4028.093	3489.538	2	4	1	6	5	3
	Std	157.2516	207.9727	56.84354	363.1331	384.0885	153.5662	3	4	1	5	6	2
*Fn*_*26*_	Best	3089.308	3090.393	3091.548	3093.179	3089.308	3095.366	1.5	3	4	5	1.5	6
	Mean	3095.819	3105.126	3095.589	3129.619	3099.07	3100.941	2	5	1	6	3	4
	Worst	3125.174	3241.286	3100.03	3209.53	3197.703	3116.125	3	6	1	5	4	2
	Std	5.586295	26.24973	1.944856	36.35269	18.70578	4.757791	3	5	1	6	4	2
*Fn*_*27*_	Best	3100	3100.001	3100	2800.009	3108.935	3182.341	2.5	4	2.5	1	5	6
	Mean	3287.389	3366.993	3191.247	3277.773	3352.853	3379.829	3	5	1	2	4	6
	Worst	3411.822	3749.371	3411.823	3412.053	3731.813	3499.317	1	6	2	3	5	4
	Std	122.8562	190.0179	88.55182	147.9899	193.3343	83.32384	3	5	2	4	6	1
*Fn*_*28*_	Best	3131.732	3132.954	3186.272	3161.176	3138.465	3153.017	1	2	6	5	3	4
	Mean	3194.966	3230.359	3223.056	3271.415	3175.727	3234.006	2	4	3	6	1	5
	Worst	3348.208	3385.623	3265.67	3424.743	3234.517	3332.945	4	5	2	6	1	3
	Std	43.33585	59.1188	16.49242	65.41059	24.26783	43.72574	3	5	1	6	2	4
							Sum	207	383.5	317	503.5	407	534
							Mean rank	1.8482	3.4241	2.8303	4.4955	3.6339	4.7678
							Final Ranking	1	3	2	5	4	6
							Improve %	–	46.02	34.7	58.88	49.14	61.2

Using Friedman ANOVA Test, Table 4 provides the P-values of the Comparisons of EGTA, GTA, DMO, SAT, RKO and AQUO. The provided p-values indicate the results of the comparisons across 28 different functions. As shown, the majority of the p-values are extraordinarily low, with many on the order of 10E−30 or even lower. This indicates highly significant differences between the compared algorithms for most of the functions. Also, the p-values range from a minimum of 1.4445E−12 to a maximum of 4.4365E−48. This wide range still falls well below the typical significance threshold (e.g., 0.05), reaffirming that all comparisons reveal significant differences. The mean p-value is 5.1896E−14, which further emphasizes the overall significant differences across the board.

**Table 4:** Friedman ANOVA test for the 28 functions.

No.	p_value	No.	p_value	No.	p_value
*Fn*_*1*_	1.0107200E-38	*Fn*_*12*_	1.0933551E-35	*Fn*_*23*_	1.4444979E-12
*Fn*_*2*_	3.9792792E-40	*Fn*_*13*_	2.0716013E-40	*Fn*_*24*_	8.5908550E-15
*Fn*_*3*_	3.5224704E-27	*Fn*_*14*_	4.4365130E-48	*Fn*_*25*_	1.7779644E-20
*Fn*_*4*_	7.6375067E-29	*Fn*_*15*_	8.9980755E-33	*Fn*_*26*_	8.4864769E-23
*Fn*_*5*_	1.5197110E-45	*Fn*_*16*_	4.2084818E-35	*Fn*_*27*_	6.8314100E-22
*Fn*_*6*_	5.9228380E-27	*Fn*_*17*_	1.3832374E-36	*Fn*_*28*_	2.7983811E-26
*Fn*_*7*_	1.3653906E-30	*Fn*_*18*_	3.3783677E-43	min(p_value)	1.4445e-12
*Fn*_*8*_	2.6790616E-42	*Fn*_*19*_	6.6402312E-40	mean(p_value)	5.1896e-14
*Fn*_*9*_	3.3716174E-33	*Fn*_*20*_	4.3444446E-29	max(p_value)	4.4365e-48
*Fn*_*10*_	4.9461919E-37	*Fn*_*21*_	8.7926582E-29		
*Fn*_*11*_	1.6540223E-40	*Fn*_*22*_	1.9469708E-20		

In order to show the computational complexity analysis for the compared methods, the “Big O analysis” [87] is implemented. Table 5 compares the computational complexity and average runtime of various algorithms (EGTA, GTA, DMO, SAT, RKO, AQUO) across 28 benchmark functions with a dimension of 10. The results indicate that all algorithms have a computational complexity of *O*(150,000), implying similar theoretical performance in terms of computational steps. However, the average runtime varies significantly. EGTA consistently shows efficient runtimes, typically around 0.3–0.4 s, demonstrating a balance between computational effort and efficiency. GTA performs similarly but slightly less efficiently than EGTA. In contrast, DMO exhibits significantly higher runtimes, often exceeding 0.5 s, making it less suitable for applications requiring quick solutions. SAT consistently has the lowest runtimes, often below 0.2 s, but this comes at the cost of solution quality as previously demonstrated. RKO and AQUO show moderate performance, with runtimes generally between 0.2 and 0.6 s, but still lag behind EGTA. Therefore, EGTA proves to be efficient and reliable, making it a preferable choice for practical applications needing quick and high-quality solutions.

**Table 5:** Computational complexity and runtime of the compare algorithms for CEC 2017 tasks.

*No.*	Dimension	Computational complexity	Average Runtime (Seconds)/Experiment					
			EGTA	GTA	DMO	SAT	RKO	AQUO
*Fn*_*1*_	10	O(150,000)	0.35554	0.37253	0.6481	0.20598	0.35374	0.53482
*Fn*_*2*_	10	O(150,000)	0.34408	0.23253	0.59179	0.0894	0.18915	0.49243
*Fn*_*3*_	10	O(150,000)	0.32689	0.21896	0.93135	0.17421	0.20869	0.46561
*Fn*_*4*_	10	O(150,000)	0.28641	0.25348	0.64058	0.10079	0.22657	0.46841
*Fn*_*5*_	10	O(150,000)	0.31134	0.3538	0.91211	0.11793	0.24685	0.49442
*Fn*_*6*_	10	O(150,000)	0.32232	0.25436	0.80667	0.11976	0.22537	0.48818
*Fn*_*7*_	10	O(150,000)	0.28874	0.3215	0.78742	0.16288	0.21571	0.56925
*Fn*_*8*_	10	O(150,000)	0.38396	0.39785	1.0543	0.11317	0.23078	0.48142
*Fn*_*9*_	10	O(150,000)	0.34235	0.28896	1.1025	0.10361	0.21796	0.49877
*Fn*_*10*_	10	O(150,000)	0.38191	0.34388	1.079	0.11772	0.22452	0.48068
*Fn*_*11*_	10	O(150,000)	0.39063	0.47725	0.88214	0.10514	0.22419	0.49107
*Fn*_*12*_	10	O(150,000)	0.3316	0.30109	0.93482	0.10478	0.21829	0.48104
*Fn*_*13*_	10	O(150,000)	0.25376	0.39291	1.0115	0.10132	0.19897	0.48849
*Fn*_*14*_	10	O(150,000)	0.28162	0.29428	1.085	0.10723	0.19063	0.6302
*Fn*_*15*_	10	O(150,000)	0.30388	0.35754	0.88598	0.099634	0.22792	0.52492
*Fn*_*16*_	10	O(150,000)	0.36239	0.522	0.84702	0.12734	0.23502	0.68648
*Fn*_*17*_	10	O(150,000)	0.34635	0.36419	0.5307	0.10284	0.24191	0.58819
*Fn*_*18*_	10	O(150,000)	0.44946	0.47736	0.68154	0.18008	0.27888	0.6625
*Fn*_*19*_	10	O(150,000)	0.26375	0.32336	0.57858	0.12473	0.22227	0.51678
*Fn*_*20*_	10	O(150,000)	0.32076	0.5172	0.54731	0.12511	0.22811	0.50726
*Fn*_*21*_	10	O(150,000)	0.3315	0.35149	0.6468	0.12609	0.23368	0.53163
*Fn*_*22*_	10	O(150,000)	0.30499	0.49695	0.55502	0.13321	0.24922	0.51529
*Fn*_*23*_	10	O(150,000)	0.30173	0.51118	0.57081	0.13465	0.24865	0.59141
*Fn*_*24*_	10	O(150,000)	0.35292	0.40677	0.709	0.19064	0.22811	0.50573
*Fn*_*25*_	10	O(150,000)	0.34998	0.37581	0.96582	0.11434	0.25724	0.53702
*Fn*_*26*_	10	O(150,000)	0.33953	0.50157	0.61161	0.13492	0.26057	0.54012
*Fn*_*27*_	10	O(150,000)	0.37754	0.36807	0.79898	0.13657	0.25169	0.52663
*Fn*_*28*_	10	O(150,000)	0.37902	0.39352	0.5649	0.14529	0.30091	0.53889

### 4.2 TCSC installations for IEEE 30-bus grid

**Fig. 3:**
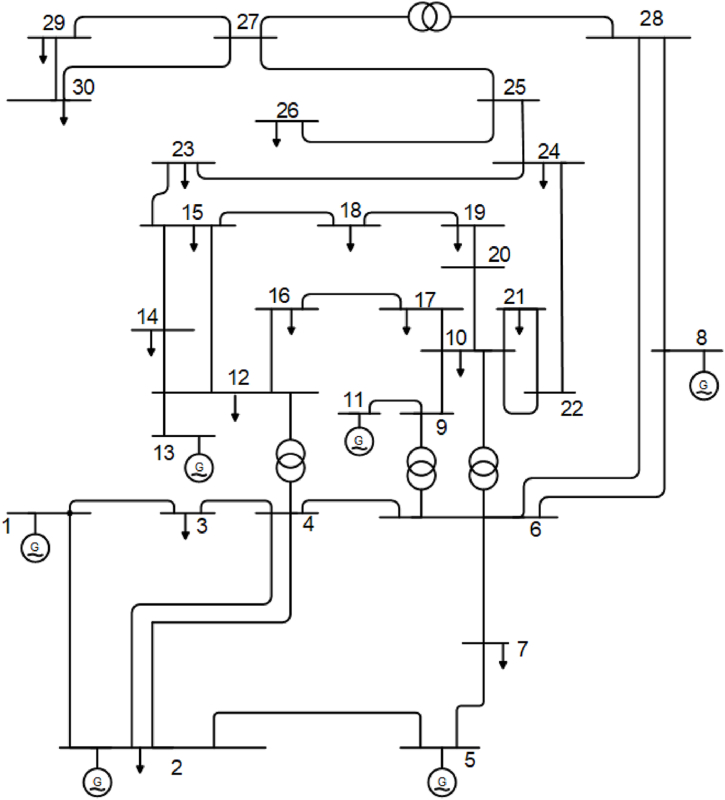
IEEE 30-bus grid [90].

In this part, the optimal TCSC allocations are managed through the IEEE standard 30-bus system, as can be seen in Fig. 3. There are 4 transformers, 41 lines, 30 nodes, and 9 compensators in this system [88,89]. The tap positions are 0.90 p.u., whilst the maximum generating voltage is 1.10 p.u. Moreover, the voltage limitations for the load buses are between 1.05 and 0.95 p.u., whilst the generator bus has limits of 1.10 and 0.90 p.u.

The proposed EGTA is compared to the original GTA as well as other modern algorithms previously applied in the literature which are AQUO [73], DMO [73], Improved DMO (IDMO) [73], Artificial Ecosystem Optimization (AEO) [91], SAT [91], Improved SAT (ISAT) [91] and Grey Wolf Optimization (GWO) [91]. The proposed novel EGTA and the original GTA are run 20 times independently, with 300 iterations and 50 searching individuals. Three distinct cases are examined, taking into account one, two, and three TCSC apparatuses, subject to the number of candidate apparatuses provided.

#### 4.2.1 Case 1: one TCSC apparatus

**Fig. 4:**
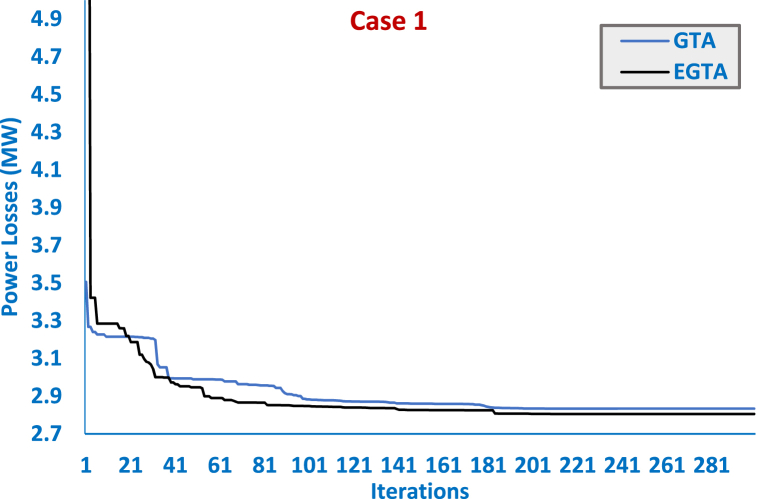
Convergence curves of EGTA versus GTA for Case 1 (IEEE 30-bus system).

In this specific instance, the innovative designed EGTA is applied for the optimization of a single TCSC device allocation, with a comprehensive comparison against the original GTA, SAT, ISAT, AEO, AQUO, and GWO detailed in Table 6. This tabulation presents essential details regarding the TCSC apparatus, encompassing location, size, and crucial control variables like generator voltage, output power, tap value, and VAr source inclusion power. Furthermore, Fig. 4 provides visual representations of the convergence trajectories for both the presented EGTA and the original GTA. Notably, the proposed EGTA exhibits outstanding performance, achieving a minimal power loss of 2.805486 MW. The algorithm identifies the transmission line (28–27) as the optimal TCSC placement, featuring a 50 % reduction in size from the installed line reactance. Remarkably, compared to the baseline scenario, the novel EGTA secures an impressive 48.10 % reduction in power losses. Moreover, the EGTA demonstrates a notable 1.01 % decrease in power losses compared to the original GTA. Additionally, when juxtaposed with AEO, SAT, ISAT, AQUO, IDMO, DMO, and GWO, the proposed EGTA showcases reductions in power losses by 1.35 %, 8.347 %, 0.575 %, 6.171 %, 0.361 %, 7.072 %, and 7.562 %, respectively. These compelling results underscore the efficiency and superiority of the EGTA in optimizing TCSC allocation, showcasing its potential for significant advancements in power grid management.

**Table 6:** Optimized controller parameters and regarding outcomes of EGTA versus GTA and other algorithms for TCSC allocations for Case 1 (IEEE 30-bus system).

	Initial Case	AEO [91]	SAT [91]	ISAT [91]	AQUO [91]	IDMO [73]	DMO [73]	GWO [73]	GTA	EGTA
VG 1	1.0500	1.099351	1.1	1.1	1.1000	1.0997	1.077325	1.099568	1.100000	1.099999984
VG 2	1.0400	1.094747	1.1	1.09755	1.1000	1.097107	1.07729	1.095818	1.100000	1.097593589
VG 5	1.0100	1.074308	1.1	1.079716	1.09728	1.078474	1.05654	1.08001	1.082023	1.079802938
VG 8	1.0100	1.083873	1.1	1.08684	1.09288	1.084998	1.066889	1.085785	1.088850	1.08694532
VG 11	1.0500	1.099959	1.1	1.1	1.1000	1.099309	1.097459	1.078706	1.100000	1.1
VG 13	1.0500	1.099709	1.1	1.1	1.1000	1.099962	1.089488	1.081997	1.100000	1.1
Ta 6–9	1.0780	1.028284	1.1	1.067173	1.1000	1.023229	0.979025	1.025412	1.070701	1.065378492
Ta 6–10	1.0690	0.925326	1.002677	0.9	0.910477	0.937607	0.94149	0.961107	0.900000	0.9
Ta 4–12	1.0320	0.999935	1.010226	0.986297	1.009181	0.983766	0.973187	1.008998	0.992874	0.987224771
Ta 28-27	1.0680	0.98665	0.978184	0.973996	1.034419	0.976997	0.968219	1.00225	0.990459	0.98094089
Qr 10	0.000	4.152465	5	5	5.000	4.453243	2.041835	2.13309	5.000000	5
Qr 12	0.000	4.930084	2.979705	5	3.962959	4.760074	3.90699	3.124115	5.000000	4.99999999
Qr 15	0.000	4.952519	4.851496	4.999997	5.000	4.095576	4.341322	0.258411	5.000000	4.866755489
Qr 17	0.000	4.912524	4.624229	4.999982	5.000	4.995081	4.602435	3.793636	5.000000	5
Qr 20	0.000	1.71465	3.910989	4.081398	5.000	4.461134	3.530735	2.796705	3.239943	4.036754825
Qr 21	0.000	4.899575	5	4.968112	5.000	4.973731	4.89273	4.209032	5.000000	5
Qr 23	0.000	0.885251	2.684319	2.58453	4.881004	2.662936	3.418359	3.763496	5.000000	2.601990955
Qr 24	0.000	3.534451	4.291999	5	5.000	4.941651	4.364901	3.481095	5.000000	4.999682937
Qr 29	0.000	2.708482	2.726024	2.275642	3.107001	2.560601	1.892259	2.864193	5.000000	2.325752153
PG 1	99.2400	51.4936	51.5016	51.21077	51.3952	51.33157	52.61437	62.3303	80.000000	80
PG 2	80.000	79.78346	80	80	80.000	79.97828	79.5501	79.61742	50.000000	50
PG 5	50.000	49.86303	49.89148	50	50.000	49.99382	49.83164	49.8189	35.000000	35
PG 8	20.000	34.99899	35	35	35.000	34.94736	34.71168	33.99505	30.000000	29.99999976
PG 11	20.000	29.55887	30	30	30.000	29.98847	29.72535	29.7921	40.000000	40
PG 13	20.000	39.98309	40	40	40.000	39.97617	39.98591	37.77225	30.000000	30
TCSC location	–	28–27	23–24	28–27	6–28	28–27	10–17	4–6	28–27	28–27
TCSC Compensation	–	−49.490 %	+4.665 %	−49.998 %	−42.017 %	−49.72 %	−11.44 %	−35.028 %	−50 %	−50 %
Losses (MW)	5.832400	2.844	3.061	2.8217	2.990	2.81565	3.019	3.035	2.833805396	**2.805486**

Positive or negative signs reveal an increase or decrease in the TCSC-connected transmission line reactance.

**Fig. 5:**
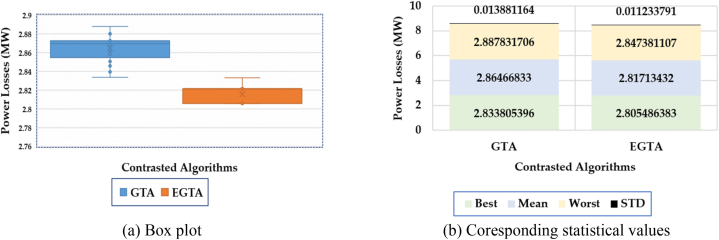
Box plot for EGTA versus GTA for Case 1 (IEEE 30-bus system).

Additionally, a comprehensive statistical assessment is conducted, and the box plot illustrating the results of both the conventional GTA and the newly proposed EGTA for Case 1 is depicted in Fig. 5. The superiority of the proposed EGTA becomes evident as it accumulates the fewest metrics from the achieved objective values. Regarding mean losses, the original GTA records losses of 2.865 MW, whereas the suggested EGTA achieves the lowest losses at 2.817 MW, showcasing an improvement of 1.6593 % over the GTA. In terms of worst-case losses, the original GTA reports losses of 2.887 MW, while the suggested EGTA attains the lowest losses at 2.847 MW, representing a 1.4 % improvement over the GTA. When considering standard deviation in losses, the original GTA exhibits losses of 0.01388 MW, whereas the suggested EGTA demonstrates the lowest losses at 0.0112 MW, indicating a notable 19.07 % improvement over the GTA. These findings underscore the consistent and superior performance of the proposed EGTA in optimizing TCSC allocation for enhanced power grid management.

#### 4.2.2 Case 2: two TCSC apparatus allocations

**Fig. 6:**
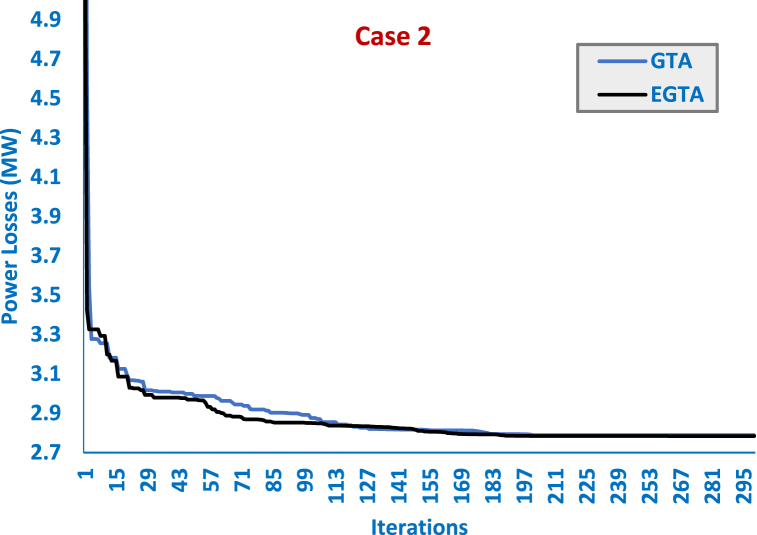
Convergence curves of EGTA versus GTA for Case 2 (IEEE 30-bus system).

In this case, the proposed EGTA is applied to optimize the allocation of two TCSC devices. Against the backdrop of the GTA, SAT, ISAT, AEO, AQUO, and GWO, the outcomes detailing the location, size of the TCSC devices, and the most suitable control variables—output power, generator's voltage, tap value, and Var source's inclusion power—are presented in Table 7. Furthermore, Fig. 6 illustrates the convergence curves for both the EGTA and the original GTA. Evidently, the suggested EGTA achieves the minimal power loss of 2.784025 MW. The proposed EGTA designates transmission lines (4–12) and (28–27) as the optimal locations for the TCSC, incorporating a 50 % addition and 50 % subtraction, respectively, from the installed line reactance. Compared to the initial scenario, the EGTA brings about a substantial 47.73 % reduction in power losses. The suggested EGTA demonstrates a noteworthy decline of 0.18 % in power losses in contrast to the original GTA. Additionally, in comparison to AEO, SAT, ISAT, AQUO, IDMO, DMO, GWO, the proposed EGTA exhibits reductions in power losses by 2.98 %, 11.42 %, 1.29 %, 7.57 %, 0.67 %, 7.98 %, 15.91 %, respectively. These results underscore the efficacy of the proposed EGTA in optimizing dual TCSC allocation for enhanced power grid efficiency.

**Table 7:** Optimized controller parameters and regarding outcomes of EGTA versus GTA and other algorithms for TCSC allocations for Case 2 (IEEE 30-bus system).

	Initial Case	AEO [91]	SAT [91]	ISAT [91]	AQUO [91]	IDMO [73]	DMO [73]	GWO [73]	GTA	EGTA
VG 1	1.0500	1.099352	1.1	1.1	1.1	1.09986	1.088672	1.095119	1.1	1.1
VG 2	1.0400	1.096829	1.1	1.097584	1.1	1.095882	1.084124	1.089415	1.1	1.097616
VG 5	1.0100	1.078726	1.1	1.079817	1.1	1.077982	1.063908	1.071614	1.082068	1.07985
VG 8	1.0100	1.086607	1.1	1.087006	1.094221	1.084631	1.076237	1.078618	1.088914	1.086999
VG 11	1.0500	1.099927	1.1	1.1	1.1	1.099862	1.098864	1.084584	1.1	1.1
VG 13	1.0500	1.099558	1.047568	1.1	1.1	1.09972	1.079165	1.074647	1.1	1.1
Ta 6–9	1.0780	0.976591	1.042502	1.064807	1.044803	1.051505	0.973169	1.049704	1.072465	1.066526
Ta 6–10	1.0690	1.016372	1.1	0.900035	0.929538	0.915569	1.004953	1.032416	0.900275	0.900009
Ta 4–12	1.0320	1.00762	1.066829	0.980145	1.011769	0.985467	0.986186	1.063337	0.987922	0.985128
Ta 28-27	1.0680	0.994596	1.066162	0.980535	1.023534	0.970748	0.974498	1.002449	0.983013	0.98147
Qr 10	0	4.482774	5	5	5	4.624782	1.238543	3.497458	5	5
Qr 12	0	3.665279	5	5	5	4.991793	3.739474	0.832066	4.999964	4.999472
Qr 15	0	3.945993	5	0	4.987603	4.625295	4.203835	4.166941	5	5
Qr 17	0	4.659533	5	5	5	4.873148	2.920776	3.173012	5	5
Qr 20	0	4.934657	5	5	4.879124	4.231455	4.001807	0.851722	5	4.011626
Qr 21	0	2.590238	5	4.999997	5	4.976453	4.127666	3.394242	5	5
Qr 23	0	2.648497	4.567337	4.274824	5	2.761689	3.891986	1.978594	2.361867	2.517684
Qr 24	0	4.935695	4.920762	5	5	4.887319	4.158987	1.815333	5	5
Qr 29	0	2.42653	3.409656	2.352175	5	2.027743	1.801884	0.977867	2.282134	2.323658
PG 1	99.2400	51.49365	51.50164	51.18843	51.39525	51.28323	53.22761	62.3303	51.1896	51.1841
PG 2	80	79.80634	80	80	80	79.94034	79.01535	72.62864	80	80
PG 5	50	49.99955	50	49.99428	50	49.9956	49.82904	49.966	50	50
PG 8	20	34.98885	35	35	35	34.9966	34.8643	32.48052	35	35
PG 11	20	29.99324	30	30	30	29.99234	29.8851	29.75128	30	30
PG 13	20	39.98501	40	40	40	39.99446	39.58471	39.47006	40	40
First TCSC	–	6–9	6–28	28.27	10–17	4–12	10–21	6–8	4–12	4–12
Compensation	–	16.10 %	3.72 %	−50.00 %	−13.64 %	49.78 %	−13.52 %	24.83 %	50 %	50 %
Second TCSC	–	4–12	23–24	6–28	6–28	2–5	15–23	16–17	27–28	27–28
Second TCSC Compensation	–	49.90 %	16.57 %	−50.00 %	−44.06 %	−25.01 %	23.10 %	−2.74 %	−50 %	−50 %
Losses (MW)	5.832400	2.867	3.102	2.820	2.995	2.802571	3.006102	3.227	2.78889199	**2.784025**

Positive or negative signs reveal an increase or decrease in the TCSC-connected transmission line reactance.

**Fig. 7:**
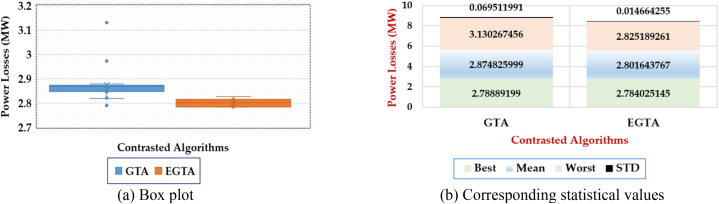
Box plot for 20 runs outcomes of EGTA versus GTA for Case 2 (IEEE 30-bus system).

In this instance, a statistical assessment is conducted, and the box plot portraying the results of both the original GTA and the innovative EGTA is depicted in Fig. 7. It's apparent that the EGTA attains superior outcomes by accumulating the fewest indices from the obtained objective values. Concerning mean incurred losses, the original GTA records losses of 2.802 MW, while the proposed EGTA identifies the lowest mean losses at 2.875 MW. For the worst incurred losses, the proposed EGTA registers the least losses at 2.825 MW, while the GTA incurs losses of 3.130 MW. Additionally, the proposed EGTA identifies the minimum standard deviation of losses at 0.0692 MW, compared to the GTA's standard deviation losses of 0.0146 MW. Against the GTA, the proposed EGTA demonstrates enhancements of 2.545 %, 9.746 %, and 78.904 % for mean, worst, and standard deviation of incurred losses, respectively.

#### 4.2.3 Case 3: three TCSC apparatus allocations

**Fig. 8:**
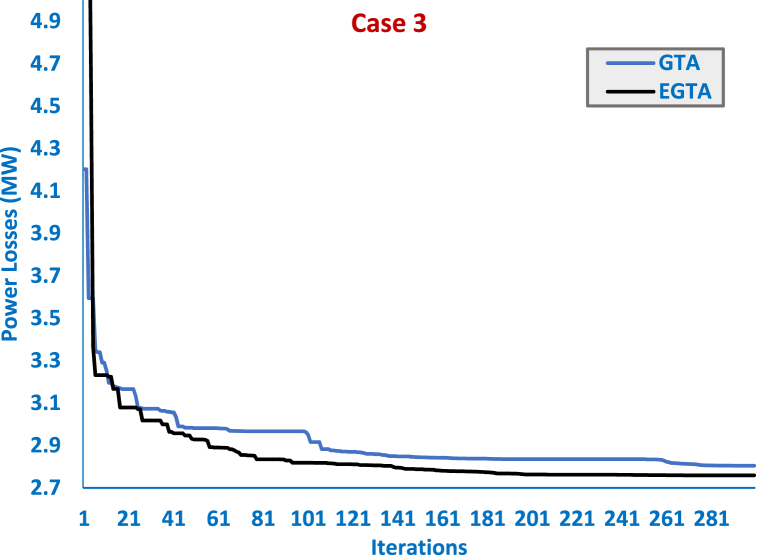
Convergence curves of EGTA versus GTA for Case 3 (IEEE 30-bus system).

In this specific scenario, three TCSC units are examined to minimize power losses. Table 8 outlines the TCSC locations, sizes, and optimal control variables—output power, generator's voltage, tap value, and Var source's inclusion power—across EGTA, GTA, SAT, ISAT, AEO, AQUO, and GWO. Additionally, Fig. 8 illustrates the convergence trends for EGTA and the original GTA. Notably, EGTA achieves the least power loss at 2.759085 MW, selecting optimal locations (4–12), (28–27), and (2–5) with specific size adjustments. The proposed EGTA attains a substantial 47.31 % reduction in power losses compared to the initial configuration, emphasizing its efficacy in optimizing TCSC allocation for enhanced power grid performance. For this case, the proposed EGTA records an improvement of 1.618 % compared to the original GTA which achieves losses of 2.804 MW. The suggested EGTA achieves a noteworthy decrease of 1.65 % in the power losses when compared to the original GTA. In addition, compared to the AEO, SAT, ISAT, AQUO, IDMO, DMO, GWO, the proposed EGTA reduces power losses by 4.38 %, 10.04 %, 2.24 %, 7.61 %, 1.29 %, 9.35 %, 15.51 %, respectively.

**Table 8:** Optimized controller parameters and regarding outcomes of EGTA versus GTA and other algorithms for Case 3 (IEEE 30-bus system).

	Initial Case	AEO [91]	SAT [91]	ISAT [91]	AQUO [91]	IDMO [73]	DMO [73]	GWO [73]	GTA	EGTA
VG 1	1.0500	1.099981	1.093023	1.1	1.097922	1.098742	1.086175	1.086747	1.1	1.1
VG 2	1.0400	1.095393	1.091724	1.1	1.097226	1.094726	1.081437	1.082197	1.097527	1.097492
VG 5	1.0100	1.076605	1.071226	1.082327	1.082092	1.076291	1.060443	1.061782	1.079819	1.079736
VG 8	1.0100	1.083436	1.088734	1.08939	1.091701	1.08014	1.065392	1.068615	1.086814	1.086674
VG 11	1.0500	1.088431	1.059316	1.1	1.095909	1.097647	1.096995	1.081636	1.1	1.1
VG 13	1.0500	1.099988	1.049747	1.1	1.088444	1.098893	1.090123	1.070184	1.1	1.1
Ta 6–9	1.0780	0.996884	1.056802	1.1	1.01364	1.044226	1.008815	0.993708	1.064124	1.063821
Ta 6–10	1.0690	0.949036	0.992325	0.9	1.045173	0.904684	0.946162	1.042592	0.9	0.900049
Ta 4–12	1.0320	1.031631	1.066913	0.990567	1.063428	0.977235	0.975652	1.018614	0.986855	0.985611
Ta 28-27	1.0680	0.976953	1.048915	0.989463	1.020301	0.979132	0.983985	0.991104	0.981415	0.980707
Qr 10	0	4.374692	5	5	5	4.844738	3.411287	2.966956	5	5
Qr 12	0	4.366721	4.893785	1.5E-06	3.430701	4.864826	2.250819	0.713832	5	5
Qr 15	0	4.974559	4.830902	5	1.754133	4.557783	2.335161	1.657207	4.747955	4.953271
Qr 17	0	0.865704	4.93972	5	4.858897	4.902339	3.443132	1.784874	5	5
Qr 20	0	2.802873	4.97807	4.40039	5	3.614843	2.022624	2.792935	3.973157	3.985859
Qr 21	0	4.07292	4.983362	5	5	4.840288	4.225295	1.804884	5	4.996885
Qr 23	0	1.849487	5	2.71585	5	3.594097	3.516276	1.079345	2.568247	2.59685
Qr 24	0	4.716259	4.999963	5	5	4.583233	4.033772	3.888447	5	5
Qr 29	0	2.050629	4.99007	2.271475	5	2.553763	4.504769	2.454247	2.57055	2.312767
PG 1	99.2400	51.43609	51.37345	51.18571	51.36892	51.57972	52.70405	56.25298	51.5603	51.1591
PG 2	80	79.97287	80	80	80	79.6883	79.32674	78.58037	80	80
PG 5	50	49.99684	50	50	50	49.93927	49.90259	49.96991	50	50
PG 8	20	34.99919	35	35	35	34.9989	34.92388	33.73529	35	35
PG 11	20	29.97878	30	30	30	29.99568	29.87712	28.5719	30	30
PG 13	20	39.89602	40	40	40	39.9928	39.68273	39.47651	40	40
1st TCSC Line	–	28–27	6–28	6–28	10–17	28–27	15–23	9–11	6–28	4–12
1st TCSC Compensation	–	−44.65 %	2.06 %	−36.96 %	−39.76 %	−48.39 %	−14.12 %	−0.62 %	50 %	50 %
2nd TCSC Line	–	6–7	–	10–20	6–28	4–12	16–17	12–13	6–28	27–28
2nd TCSC Compensation	–	−5.97 %	–	−50.00 %	6.83 %	45.30 %	−14.06 %	−7.28 %	−38.416 %	−50 %
3rd TCSC Line	–	10–20	–	28–27	25–26	6–7	23–24	–	27–28	2–5
3rd TCSC Compensation	–	−49.50 %	–	−50.00 %	−50.00 %	47.00 %	−37.21 %	–	−49.99 %	−24.847 %
Losses (MW)	5.832400	2.880	3.036	2.821	2.969	2.794672	3.017108	3.187	2.804478	**2.759085**

Positive or negative signs reveal an increase or decrease in the TCSC-connected transmission line reactance.

**Fig. 9:**
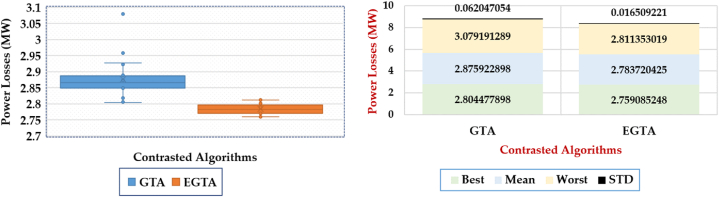
Box plot for 20 Runs Outcomes of EGTA versus GTA for Case 3 (IEEE 30-bus system).

In a statistical assessment, the box plot in Fig. 9 illustrates outcomes for both the original GTA and the proposed EGTA in Case 3. The EGTA excels with minimal indices, showcasing superior results. For mean losses, the original GTA records 2.876 MW, while EGTA achieves the lowest at 2.784 MW. Compared to GTA, EGTA consistently outperforms, attaining the least losses at 2.811 MW for worst-case scenarios, while GTA registers 3.079 MW in losses. This highlights EGTA's enhanced performance, showcasing improved reductions in worst-case losses against GTA's outcomes.

#### 4.2.4 Voltage enhancement with EGTA-Driven TCSC installations in IEEE 30-bus system

**Fig. 10:**
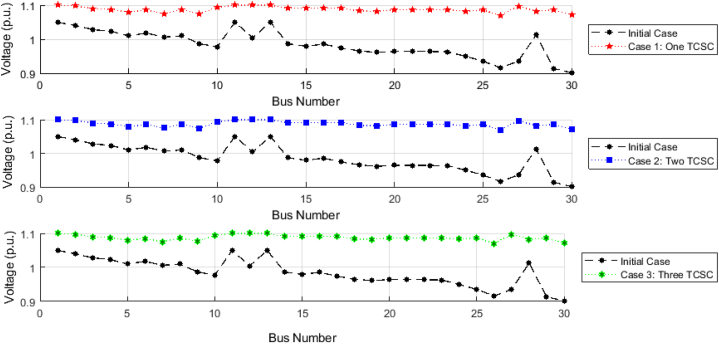
Voltage profile using EGTA following potential TCSC installation (IEEE 30-bus system).

The voltage profiles of grid buses resulting from the application of the proposed EGTA and the integration of TCSC in three distinct cases are illustrated in Fig. 10, providing a comparative analysis with the initial case. Notably, the voltage levels at various grid buses showcase significant improvements across these scenarios. The most pronounced enhancement is observed at the terminal grid bus (No. 30), where noteworthy improvements of 15.77 %, 15.75 %, and 15.79 % are realized for Cases 1, 2, and 3, respectively, marking a transition from 0.9012 per unit (p.u.) to 1.0699, 1.0697, and 1.0702 p.u. Averaging across the entire system, the mean voltage value of 0.9842 p.u. in the initial scenario undergoes a considerable 9.5 %, 9.49 %, and 9.51 % enhancement with the incorporation of one, two, and three TCSC installations, respectively.

### 4.3 TCSC installations for IEEE 57-bus grid

**Fig. 11:**
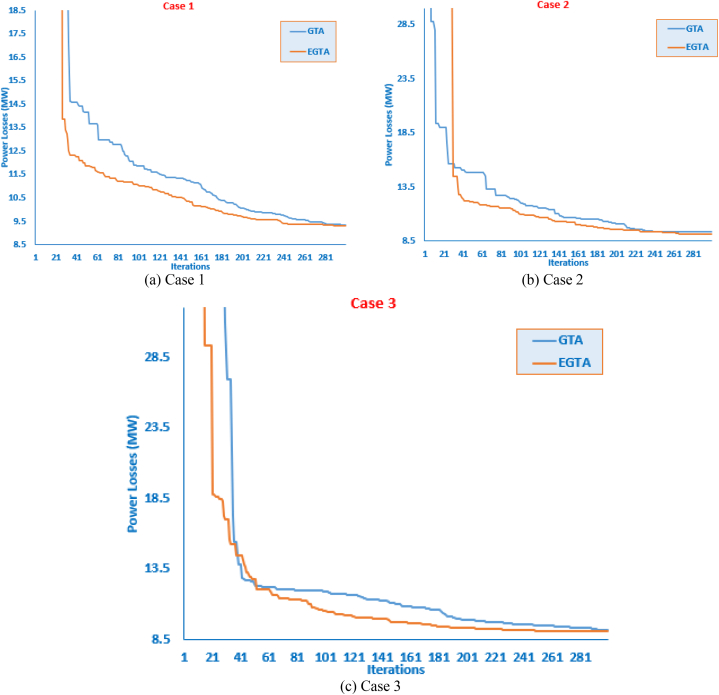
Convergence curves of EGTA versus GTA for Cases 1–3 (IEEE 57-bus system).

This part uses the standard IEEE 57-bus transmission network. There are 57 nodes, 80 lines, 7 generators, 17 on-load tap changing transformers, and 3 capacitive sources on buses in this system [92]. In order to achieve minimum power losses, the three situations under study are examined with consideration for one, two, and three TCSC apparatuses. Table 9 tabulates their obtained control variables, the EGTA and GTA are applied. Alternatively, Fig. 11 reveals the regarding converging features of the proposed EGTA and the original GTA.

**Table 9:** Optimized controller parameters and regarding outcomes of EGTA versus GTA for TCSC apparatus allocations concerning Cases 1–3 (IEEE 57-bus system).

	Initial Scenario	Case 1		Case 2		Case 3	
		GTA	EGTA	GTA	EGTA	GTA	EGTA
Vg _1_	1.010000	1.097491	1.099989	1.099995	1.093567	1.098687	1.093502
Vg _2_	1.010000	1.092158	1.094947	1.093151	1.089039	1.092811	1.086751
Vg _3_	1.010000	1.096217	1.099988	1.093494	1.095684	1.094534	1.08687
Vg _6_	1.010000	1.098428	1.1	1.090891	1.096776	1.095182	1.085401
Vg _8_	1.010000	1.099598	1.1	1.09306	1.099975	1.096474	1.088437
Vg _9_	1.010000	1.081689	1.084081	1.078383	1.082019	1.079246	1.073016
Vg _12_	1.010000	1.083189	1.093116	1.089058	1.085935	1.086006	1.082908
Tap _4-18_	0.970000	1.067685	1.093321	1.099999	0.976586	1.1	1.071967
Tap _4-18_	0.978000	1.077534	1.074644	0.994462	1.021982	1.06852	0.946759
Tap _21-20_	1.043000	0.996154	1.034112	1.09999	0.994251	1.072312	0.999371
Tap _24-25_	1.000000	1.099877	1.077278	1.097604	1.1	1.024114	1.08994
Tap _24-25_	1.000000	1.1	0.9	1.054016	1.019173	1.077488	0.966074
Tap _24-26_	1.043000	0.988617	1.022207	1.00733	1.025354	0.994138	1.007865
Tap _7-29_	0.967000	0.996103	1.053759	0.984974	1.015318	0.988853	0.979044
Tap _34-32_	0.975000	0.964743	0.923615	1.054399	0.963629	0.977272	0.961624
Tap _11-41_	0.955000	0.922148	0.9	1.078596	0.98221	0.900699	1.005919
Tap _15-45_	0.955000	0.991501	1.00942	0.980001	0.973958	0.997985	0.972695
Tap _14-46_	0.900000	0.999395	1.013107	0.966496	0.973046	0.99782	0.965377
Tap _10-51_	0.930000	1.003225	1.023517	0.974979	0.990973	0.988828	0.967784
Tap _13-49_	0.895000	0.982207	1.000989	0.939942	0.970916	0.972732	0.957129
Tap _11-43_	0.958000	0.984627	0.982672	0.952465	0.962005	0.969907	0.950563
Tap _40-56_	0.958000	0.988673	0.960803	1.05717	0.992833	0.999632	1.02016
Tap _39-57_	0.980000	0.94573	0.932103	1.019128	0.990643	0.949028	1.005477
Tap _9-55_	0.940000	0.997169	1.046267	0.978617	1.012098	0.994473	0.976605
Qc _18_	10.000000	29.08747	17.43302	2.74E-06	12.11104	15.9771	14.43164
Qc _25_	5.900000	18.05743	15.46386	16.74205	16.73376	17.15023	15.34241
Qc _53_	6.300000	21.16867	14.1444	12.72448	15.20355	13.7651	10.87857
Pg _1_	478.635000	203.3409	201.3377	205.9872	199.7322	197.2383	206.5935
Pg _2_	0.000000	1.32E-11	6.21E-26	3.63E-09	4.54E-07	4.488473	8.37E-15
Pg _3_	40.000000	140	140	139.9993	139.9996	139.9996	137.5864
Pg _6_	0.000000	99.99684	99.95308	99.99933	99.43924	99.99855	100
Pg _8_	450.000000	306.8097	308.8158	304.1783	310.7573	308.2217	305.6717
Pg _9_	0.000000	100	100	99.99914	99.99068	100	100
Pg _12_	310.000000	410	409.9986	409.9988	410	409.9996	410
First TCSC installed Lines	–	13–49	13–49	9–55	9–55	9–55	1–15
First TCSC Compensation	–	−50.00 %	−49.64 %	−14.25 %	−17.68 %	50.00 %	−19.63 %
Second TCSC installed Lines	–	–	–	1–15	13–49	13–49	38–48
Second TCSC Compensation	–	–	–	−22.70 %	−50.00 %	−50.00 %	−33.37 %
Third TCSC installed Lines	–	–	–	–	–	–	13–49
Third TCSC Compensation	–	–	–	–	–	–	−48.78 %
Losses (MW)	27.835	9.347426	9.305192	9.362048	9.118985	9.146321	9.051817

Positive or negative signs reveal an increase or decrease in the TCSC-connected transmission line reactance.

As shown in Table 9, for the first case, the proposed EGTA identifies the transmission line (13–49) as the optimal TCSC placement, featuring a 49.64 % reduction in size from the installed line reactance. Also, it sets the other control variables simultaneously which reduces the power losses from 27.835 MW in the initial case to 9.3052 MW while the GTA achieves counterpart losses of 9.347 MW. Remarkably, compared to the baseline scenario, the developed EGTA secures an impressive 66.55 % reduction in power losses. Moreover, the EGTA demonstrates a notable 0.449 % decrease in power losses compared to the original GTA.

In the second scenario, the EGTA optimization identifies the optimal TCSC placements at transmission lines (9–55) and (13–49), resulting in a significant 17.68 % and 50 % reduction in size from the installed line reactance. Additionally, it achieves a reduction in power losses to 9.119 MW, surpassing the GTA's losses of 9.362 MW. Notably, the developed EGTA showcases a substantial 2.6 % decrease in power losses compared to the original GTA.

Moving to the third case, the proposed EGTA identifies optimal TCSC placements at transmission lines (1–15), (38–48), and (13–49), showcasing substantial reductions in size by 19.63 %, 33.37 %, and 48.78 % from the installed line reactance. Moreover, it achieves a reduction in power losses to 9.051 MW, while the GTA experiences counterpart losses of 9.1463 MW. Thus, the developed EGTA demonstrates a noteworthy 1.042 % decrease in power losses compared to the original GTA.

**Fig. 12:**
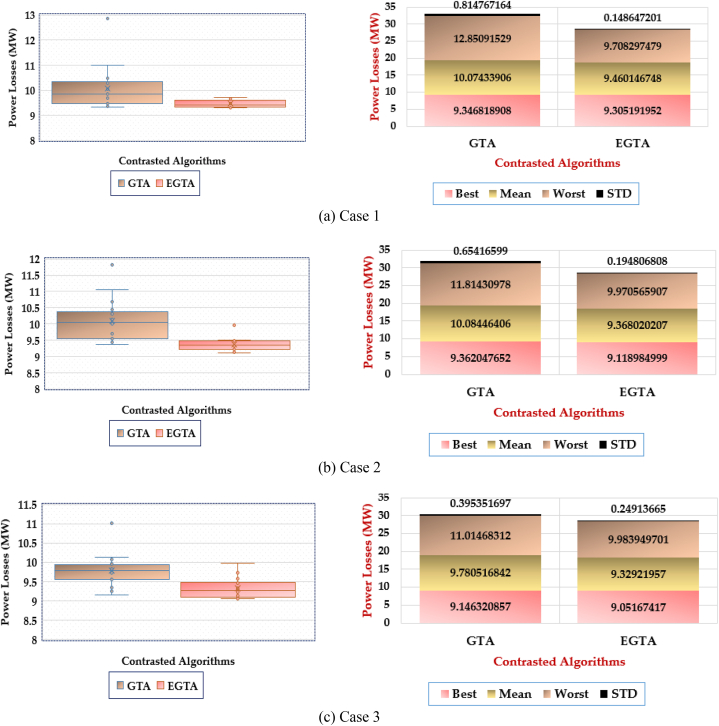
Box plot for 20 Runs Outcomes of the EGTA versus GTA Cases 1–3 (IEEE 57-bus system).

Moreover, Fig. 12 presents a box plot illustrating the outcomes of the proposed EGTA and the original GTA. It is evident that the EGTA outperforms by accumulating fewer indices among the obtained objective values. The implications from this figure are as follows.

• In the first scenario, the proposed EGTA identifies the lowest losses at 9.4601 MW based on mean acquired losses, while the original GTA records mean losses of 10.0743 MW. Thus, in comparison to the original GTA results, the proposed EGTA exhibits improvement reductions of 6.096 %.
• In the second scenario, the proposed EGTA identifies the lowest losses at 9.368 MW based on mean acquired losses, while the original GTA records mean losses of 10.0844 MW. Consequently, in comparison to the original GTA results, the proposed EGTA demonstrates improvement reductions of 7.107 %.
• In the third scenario, the proposed EGTA identifies the lowest losses at 9.3292 MW based on mean acquired losses, while the original GTA records mean losses of 9.781 MW. Thus, in comparison to the original GTA results, the proposed EGTA displays improvement reductions of 4.62 %.

**Fig. 13:**
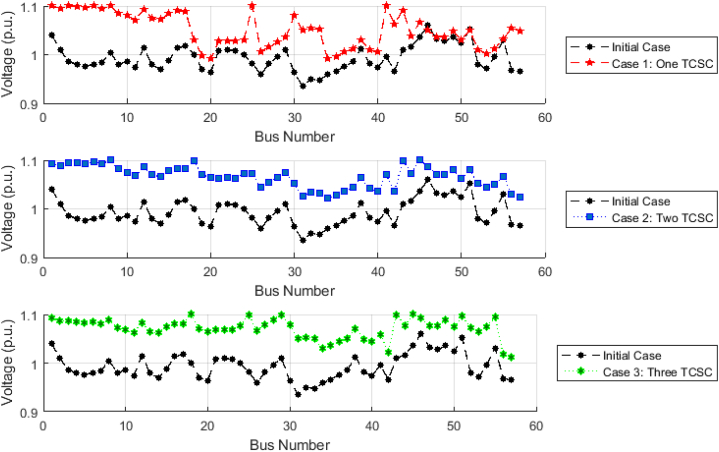
Voltage profile using EGTA following potential TCSC installation (IEEE 57-bus system).

For the IEEE-57 bus system, the voltage profiles of grid buses resulting from the application of the proposed EGTA and the integration of TCSC in three distinct cases are illustrated in Fig. 13, providing a comparative analysis with the initial case. Notably, the voltage levels at various grid buses showcase significant improvements across these scenarios. The most pronounced enhancement is observed at the terminal grid bus (No. 31), where noteworthy improvements of 5.58 %, 8.42 %, and 7.52 % are realized for Cases 1, 2, and 3, respectively, marking a transition from 0.9353 per unit (p.u.) to 0.9912, 1.0219, and 1.012 p.u.

Therefore, the application of more TCSC devices in this system with the EGTA implementation helps in achieving better voltage profile. Specifically, in terms of average voltages across the entire system, the initial mean value of 0.9942 p.u. experiences a substantial 4.15 %, 5.79 %, and 6.65 % enhancement with the introduction of one, two, and three TCSC installations, respectively. This results in a transition from 0.994217 per unit (p.u.) to 1.03725, 1.05536, and 1.064993 p.u., signifying a noteworthy improvement in voltage levels.

Table 10 presents a comparative analysis of power losses (MW) for different algorithms applied to the IEEE 57-bus system. It compares the performance of the proposed EGTA and the original GTA against two published methods: DMO and IDMO from Ref. [73]. In Case 1, the power losses for DMO and IDMO are 13.30243 MW and 9.846252 MW, respectively. The GTA reduces these losses to 9.347426 MW. However, the EGTA further reduces the losses to 9.305192 MW, achieving the lowest power loss among the compared algorithms. For Case 2, the power losses recorded by DMO and IDMO are 13.00813 MW and 9.951942 MW, respectively. The GTA achieves a lower loss of 9.362048 MW, but the EGTA outperforms all other methods with a further reduced loss of 9.118985 MW. In Case 3, the power losses for DMO and IDMO are 13.20578 MW and 9.746247 MW, respectively. GTA reduces the losses to 9.146321 MW, and once again, EGTA achieves the lowest power losses with 9.051817 MW. This consistent reduction across all cases underscores the effectiveness of the EGTA in optimizing TCSC allocation. The incremental improvements from GTA to EGTA demonstrate the significant benefits brought by the enhancements introduced in the EGTA. These comparisons validate the improvements made in the EGTA, demonstrating its superior capability in optimizing TCSC allocation and improving power grid performance.

**Table 10:** Comparisons of EGTA versus GTA with published results for IEEE 57-bus system.

Case Study	Algorithms	Losses (MW)
Case 1	DMO [73]	13.30243
	IDMO [73]	9.846252
	GTA	9.347426
	EGTA	9.305192
Case 2	DMO [73]	13.00813
	IDMO [73]	9.951942
	GTA	9.362048
	EGTA	9.118985
Case 3	DMO [73]	13.20578
	IDMO [73]	9.746247
	GTA	9.146321
	EGTA	9.051817

## 5 Conclusion

This study introduces the Enhanced Gorilla Troops Algorithm (EGTA), an advanced metaheuristic designed to address the optimization challenges associated with Thyristor Controlled Series Capacitors (TCSC) allocation in power grids. By enhancing the standard Gorilla Troops Algorithm (GTA) with adaptive mechanisms, EGTA effectively mitigates common metaheuristic issues such as premature convergence and local optima entrapment. Extensive benchmarking on the CEC suite and the TCSC allocation problem demonstrates that EGTA significantly outperforms GTA and other state-of-the-art algorithms in terms of convergence speed and solution quality. The statistical validation of our results using the Friedman ANOVA test further confirms the robustness and effectiveness of EGTA, with highly significant p-values across 28 different functions. The practical application of EGTA to optimize TCSC allocation highlights its utility in real-world engineering problems, showcasing its potential to enhance the efficiency and stability of power systems. This study not only advances the theoretical understanding of metaheuristic optimization but also provides a robust and efficient tool for practical applications.

### 5.1 Advantages and disadvantages

The EGTA offers several advantages over existing optimization algorithms. Firstly, it demonstrates improved optimization performance, outperforming the standard GTA and other state-of-the-art methods in terms of convergence speed and solution quality. This makes it highly effective for tackling complex optimization problems. Additionally, EGTA incorporates adaptive mechanisms that strike a balance between exploration and exploitation, reducing the risk of premature convergence and enhancing its ability to find global optima. The algorithm's robustness is supported by statistical validation, showing significant differences in performance. Furthermore, EGTA has proven its practical applicability by successfully optimizing TCSC in power grids, highlighting its potential to enhance power system efficiency and stability. Its flexibility allows for handling multi-objective optimization and adapting to dynamic scenarios, making it a versatile tool for various applications. However, there are certain disadvantages to consider. The computational complexity of EGTA can be higher compared to simpler algorithms due to its adaptive mechanisms and enhanced features, leading to longer computation times for large-scale problems. Parameter sensitivity is another challenge, as finding optimal settings may require additional tuning and experimentation. Implementing EGTA may also be more complex, necessitating a deeper understanding of the algorithm's inner workings. Lastly, the effectiveness of EGTA can depend on the specific problem characteristics, which may require problem-specific adjustments for optimal performance.

### 5.2 Future research directions

Future research will focus on further enhancing the performance of EGTA and expanding its applicability within the power system field. Applying EGTA to dynamic optimization problems in power systems can be extended, such as real-time load balancing, dynamic pricing, and adaptive network reconfiguration. Also, extending EGTA to handle multi-objective optimization problems in power systems can be handled, including the simultaneous optimization of power loss reduction, voltage stability, and cost minimization. Additionally, utilizing EGTA to optimize the integration of renewable energy sources into power grids can be performed, addressing challenges such as optimal placement and sizing of distributed generation units, and managing the variability and uncertainty of renewable energy production. Moreover, exploring the application of EGTA in smart grid environments, focusing on optimizing demand response programs, enhancing grid resilience, and improving the efficiency of distributed energy resources management.

## CRediT authorship contribution statement

**Mohammed H. Alqahtani:** Writing – review & editing, Validation, Supervision, Resources, Project administration, Investigation, Funding acquisition, Methodology, Formal anaylsis. **Sulaiman Z. Almutairi:** Writing – review & editing, Visualization, Validation, Software, Resources, Investigation, Data curation. **Ali S. Aljumah:** Writing – original draft, Visualization, Validation, Software, Investigation, Conceptualization. **Ahmed R. Ginidi:** Writing – review & editing, Writing – original draft, Visualization, Software, Methodology, Investigation, Formal analysis, Conceptualization. **Abdullah M. Shaheen:** Writing – review & editing, Writing – original draft, Validation, Software, Methodology, Formal analysis, Conceptualization.

## Declaration of competing interest

The authors declare that they have no known competing financial interests or personal relationships that could have appeared to influence the work reported in this paper.
